# FAM3C‐YY1 axis is essential for TGFβ‐promoted proliferation and migration of human breast cancer MDA‐MB‐231 cells via the activation of HSF1

**DOI:** 10.1111/jcmm.14243

**Published:** 2019-03-19

**Authors:** Weili Yang, Biaoqi Feng, Yuhong Meng, Junpei Wang, Bin Geng, Qinghua Cui, Hongquan Zhang, Yang Yang, Jichun Yang

**Affiliations:** ^1^ Key Laboratory of Molecular Cardiovascular Sciences of the Ministry of Education, Department of Physiology and Pathophysiology, School of Basic Medical Sciences Center for Non‐coding RNA Medicine, Peking University Health Science Center Beijing China; ^2^ Key Laboratory of Molecular Cardiovascular Sciences of the Ministry of Education, Department of Biomedical Informatics, School of Basic Medical Sciences Center for Non‐coding RNA Medicine, Peking University Health Science Center Beijing China; ^3^ State Key Laboratory of Cardiovascular Disease Hypertension Center, Fuwai Hospital, Peking University Health Science Center, CAMS & PUMC Beijing China; ^4^ Key Laboratory of Carcinogenesis and Translational Research (Ministry of Education), and State Key Laboratory of Natural and Biomimetic Drugs Peking University Health Science Center Beijing China; ^5^ Department of Biochemistry and Molecular Biology, School of Basic Medical Sciences Peking University Health Science Center Beijing China

**Keywords:** breast cancer, FAM3C, HSF1, YY1

## Abstract

Family with sequence similarity three member C (FAM3C) (interleukin‐like EMT inducer [ILEI]), heat shock factor 1 (HSF1) and Ying‐Yang 1 (YY1) have been independently reported to be involved in the pathogenesis of various cancers. However, whether they are coordinated to trigger the development of cancer remains unknown. This study determined the role and mechanism of YY1 and HSF1 in FAM3C‐induced proliferation and migration of breast cancer cells. In human MDA‐MB‐231 breast cancer cell line, transforming growth factor‐β (TGFβ) up‐regulated FAM3C, HSF1 and YY1 expressions. FAM3C overexpression promoted the proliferation and migration of MDA‐MB‐231 cells with YY1 and HSF1 up‐regulation, whereas FAM3C silencing exerted the opposite effects. FAM3C inhibition repressed TGFβ‐induced HSF1 activation, and proliferation and migration of breast cancer cells. YY1 was shown to directly activate HSF1 transcription to promote the proliferation and migration of breast cancer cells. YY1 silencing blunted FAM3C‐ and TGFβ‐triggered activation of HSF1‐Akt‐Cyclin D1 pathway, and proliferation and migration of breast cancer cells. Inhibition of HSF1 blocked TGFβ‐, FAM3C‐ and YY1‐induced proliferation and migration of breast cancer cells. YY1 and HSF1 had little effect on FAM3C expression. Similarly, inhibition of HSF1 also blunted FAM3C‐ and TGFβ‐promoted proliferation and migration of human breast cancer BT‐549 cells. In human breast cancer tissues, FAM3C, YY1 and HSF1 protein expressions were increased. In conclusion, FAM3C activated YY1‐HSF1 signalling axis to promote the proliferation and migration of breast cancer cells. Furthermore, novel FAM3C‐YY1‐HSF1 pathway plays an important role in TGFβ‐triggered proliferation and migration of human breast cancer MDA‐MB‐231 cells.

## 1 INTRODUCTION

Breast cancer is one malignant tumour that occurs in the breast epithelium. It was reported that breast cancer had become the most common cancer in the female population worldwide in 2012.^1^ In 2012, about 520 000 people died of breast cancer worldwide.^1^ In China, breast cancer is the most common cancer and accounts for about 15% of newly diagnosed cancers in women.^2^ It was estimated that there would be 2.5 million breast cancer patients in China by 2035.^2^ Clearly, breast cancer has become a severe disease endangering the health of women worldwide. More intensive studies are needed to probe the mechanisms of breast cancer.

Transforming growth factor‐β (TGFβ) protein has dual roles in the development of tumorigenesis. It inhibits tumour growth by inducing cell cycle arrest and apoptosis in the early stage of tumour formation.^3^, ^4^ However, with the progression of tumour, TGFβ stimulates the proliferation and migration of cancer cells, promoting tumour growth and invasion.^3^, ^4^ Protein kinase B (Akt) is an important intracellular molecule that stimulates cell proliferation and inhibits apoptosis^5^, ^6^ beyond its roles in regulating glucose and lipid metabolism as the key molecule of insulin signalling transduction.^7^, ^8^ It has been reported that Akt activation plays a critical role in TGFβ‐promoted development of breast cancer.^9^, ^10^

Family with sequence similarity 3 (FAM3) cytokine‐like gene family discovered in 2002 consists of four members designated as FAM3A, FAM3B, FAM3C and FAM3D, respectively.^13^ It has been reported that FAM3C is involved in TGFβ‐induced epithelial‐mesenchymal transition (EMT),^14^ and it is also called interleukin‐like EMT inducer (ILEI). Knockdown of FAM3C inhibits TGFβ‐induced EMT.^14^ So far, although FAM3C expression has been reported to be increased in various cancer tissues including breast cancer tissue,^14^, ^15^ its mechanism(s) in tumour cell proliferation and migration still remains largely unknown. Particularly, the role of FAM3C in TGFβ‐induced Akt activation and cell proliferation/migration in breast cancer cells remains unrevealed. Heat shock factor 1 (HSF1) is a transcription factor controlling the process of stress, shock response, development and many other biological processes by inducing the expression of heat shock proteins (HSPs).^21^, ^22^ Heat shock factor 1 is also activated in breast cancer tissue, and its high expression level is associated with poor prognosis of breast cancer.^23^, ^24^ Clearly, both FAM3C and HSF1 are important biomarkers and potential targets for the diagnosis and treatment of breast cancer. However, whether they are coordinated to promote the development of breast cancer remains unknown. It has been established that FAM3A, FAM3B and FAM3C of FAM3 gene family are important modulators of Akt activity via different pathways in hepatocytes.^26^ We had recently demonstrated that FAM3C activates HSF1 to induce calmodulin 1 (CALM1) gene transcription, elevating calmodulin (CaM) protein level to activate Akt pathway and repress gluconeogenesis and lipogenesis independent of insulin and calcium in hepatocytes.^27^, ^28^ However, how FAM3C activates HSF1 transcription remains unrevealed in hepatocytes in our previous studies.^27^, ^28^ Collectively, these previous findings had raised an important hypothesis that FAM3C may activate HSF1‐Akt pathway to promote breast cancer growth.

This study aimed to determine whether and how FAM3C activated HSF1 transcription to promote the proliferation and migration of breast cancer cells. Furthermore, the role of FAM3C‐HSF1 pathway in TGFβ‐induced proliferation and migration of breast cancer cells would also be studied.

## 2 EXPERIMENTAL PROCEDURE

### 2.1 Cell culture and treatment

Human breast cancer cell lines MDA‐MB‐231 cells were cultured in DMEM (Gibco, Waltham, MA, USA) and BT‐549 cells were cultured in Roswell Park Memorial Institute medium 1640 (Gibco) supplemented with 10% foetal bovine serum (FBS) (Hyclone, Pittsburgh, PA, USA), 2 mmol/L L‐glutamine, 100 units/mL penicillin (North China Pharmaceutical Co. Ltd, China, Shijiazhuang, China) and 100 units/mL streptomycin (North China Pharmaceutical Co. Ltd). All cells were cultured at 37°C in a humidified atmosphere consisting of 5% CO_2_ and 95% air. The cells were treated with TGFβ (PeproTech, Rocky Hill, NJ, USA; 2 or 5 ng/μL) for 24 hours before analysis. Cells were infected with 25 MOI Ad‐GFP or Ad‐FAM3C for 12 hours in the absence or presence of KRIBB11 (Selleck Chemicals, Shanghai, China, 10 μmol/L)^27^, ^28^ before experimental assays. For FAM3C and YY1 knockdown, cells were treated with 50 nmol/L small interfering RNA (siRNA) mixture against human FAM3C or YY1 mRNA (Beijing Biolino Nucleic Acid Technology Co., Ltd, Beijing, China, the same concentration of scrambled siRNAs was used as negative control) for 12 hours, followed by the treatment with Ad‐FAM3C or 2 ng/µL TGFβ for 12 hours. All siRNAs used in this study are naked siRNA, and the siRNA sequences were listed in Table S1.

### 2.2 Western blotting assays

Proteins were extracted from cells using Roth lysis buffer containing fresh protease and phosphatase inhibitors (Applygen, Beijing, China). Cell lysates were centrifuged at 13 680 *g* for 10 minutes at 4°C. Protein contents in the supernatant were quantified using bicinchoninic acid (BCA) Protein Assay Kit (Thermo scientific, Waltham, MA, USA). Protein samples were separated by SDS‐PAGE and transferred to a nitrocellulose membrane. Immunoblotting was conducted using primary antibodies against target genes. After overnight incubation with primary antibodies, membranes were washed and incubated with horseradish peroxidase‐conjugated secondary antibodies (Biodragon, Beijing, China) and then were detected using chemiluminescence kit (Santa Cruz Biotechnology, Santa Cruz, CA, USA). Glyceraldehyde‐3‐phosphate dehydrogenase (GAPDH) was analysed using a rabbit polyclonal as loading control. Anti‐FAM3C antibody was purchased from Abcam (ab72182; Cambridge, UK), antibodies against phosphorylated Akt (pAkt) (Ser473) (4060S), Akt (9272S), HSF1 (4356S) and Cyclin D1 (2922S) were purchased from Cell Signalling Technology Inc (Danvers, MA, USA). Anti‐YY1 antibody (66281‐1‐Ig) was purchased from Proteintech (Wuhan, China). GAPDH antibody (TA08) was purchased from Beijing Zhong Shan‐Golden Bridge Biological Technology Co., Ltd (Beijing, China). The dilutions of antibodies were 1:1000 with 5% bovine serum albumin (BSA) for Western blotting assays and 1:100 with 1% BSA for immunohistochemical staining assays.

### 2.3 Real‐time PCR assays

Total RNA (3‐5 μg) isolated from cultured cells was converted to cDNA using cDNA synthesis kit (Thermo scientific) following the manufacturer's standard protocol. The protocol for real‐time PCR analysis is as following: 95°C for 5 minutes, followed by 40 cycles at 95°C for 30 seconds, 59°C for 30 seconds and 72°C for 30 seconds. The Cycle threshold (Ct) values for the targets and GAPDH genes were provided by real‐time PCR instrumentation. The comparative method 2^−ΔΔCt^ was used for the relative quantification of target gene transcription between the control and the treated groups.^27^, ^28^ All primer sequences for real‐time PCR assays were listed in Table S2.

### 2.4 Cell counting by haemocytometer

After treatments, the cells were split and resuspended in culture medium. The cell suspension was thoroughly mixed and the cells were dispersed. A small amount of sample was drawn from the groove on both sides of the middle platform of the haemacytometer. The haemocytometer was placed on the stage of the microscope and clamped. The cell numbers were counted.

### 2.5 Plasmid transfection

One day before transfection, an appropriate amount of MDA‐MB‐231 cells were seeded in a six‐well plate. When the cells were about 70% confluence, they were transfected with HSF1 or YY1 plasmid with VigoFect transfection reagent (Vigorous Technology, Beijing, China). After 12 hours, morphological observation, cell counting and other experiments were performed. The mRNA and protein levels were analysed as above. Heat shock factor 1 plasmid expressing human HSF1 gene was purchased from OriGene^27^ (HSF1, Cat No RG200314, in pCMV6‐AC‐GFP vector, Rockville, MD, USA) and YY1 plasmid (in pCDNA3.1 vector) expressing mouse YY1 gene was kindly provided by Prof. Yan Lu of Fudan University, China.

### 2.6 Cell viability assay

Cell viability was determined as detailed previously using 3‐(4,5‐Dimethylthiazol‐2‐yl)2,5‐diphenyl tetrazolium bromide (MTT) (VETEC, Shanghai, China) methodology.^6^ MTT assays were performed as detailed previously.^6^ The values were normalized to that of control groups of cells.

### 2.7 Cell migration assays

Cell motility was assessed using a wound healing assay. Treated cells were wounded by a 200 μL plastic pipette tip, and washed using phosphate‐buffered saline (PBS) to remove cellular debris. After 0 and 12 hours, images of the wound areas under each condition were photographed. Migration rate was calculated by measuring the move distance of cells as detailed previously.^6^ Moreover, the rate of wound healing was also evaluated by calculating wound area as detailed elsewhere^29^, ^30^ using ImageJ software (http://rsb.info.nih.gov/nih-image/). In the later method, the wound closure rate was determined using the formula: wound closure rate (%) = ([wound area_0h_ − wound area_12h_]/wound area_0h_) × 100.

### 2.8 Transwell migration assay

The method for transwell migration assay was detailed elsewhere.^6^, ^31^ In brief, MDA‐MB‐231 cells treated with different conditions for 24 hours were seeded into the upper transwell chambers (Corning, Lot#3422) containing DMEM medium without FBS (30 000 cells seeded per chamber). The chambers were inserted into each well of 24‐well plates containing 600 μL DMEM medium with 10% FBS. The cells were then incubated for 12 hours. The cells that migrated through to the other side of the chamber were stained with a crystal violet staining solution. The crystal violet was dissolved in 33% ethanoic acid, and the absorbance was determined at 570 nm. The data were normalized to the control value.

### 2.9 Cell cycle detection assay

When the cells reached 60%‐80% confluence, cells were treated with siRNA, Ad‐FAM3C or TGFβ for 24 hours. The cells were collected and washed twice with PBS, fixed with 70% ethanol for 1 hour at 4°C, washed again with PBS, and re‐suspended with propidium iodide (PI) solution (0.05 mg/mL) containing RNase. Next, they were incubated at room temperature in the dark for 30 minutes. The DNA content was analysed using a flow cytometer.

### 2.10 Luciferase reporter assay

The human HSF1 gene promoter fragment flanking −2000 to 0 bp cloned from MDA‐MB‐231 cells was inserted into the pGL3‐basic vector. The positive clone was selected and confirmed by DNA sequencing. The method for promoter activity assay was detailed previously.^8^ The pHSF1 promoter‐firefly luciferase and pRL‐TK‐ranilla luciferase was cotransfected with YY1 plasmid or GFP plasmid into MDA‐MB‐231 cells using VigoFect transfection reagent (Vigorous Biotechnology, Beijing, China). After 12 hours, replace the culture supernatant with fresh medium. The activation of firefly luciferase and ranilla luciferase were measured with the Dual‐Luciferase reporter assay kit (Promega, Madison, WI, USA) 24 hours after transfection. The data in each read were normalized by the data of ranlila luciferase.

### 2.11 Immunohistochemistry

Three breast cancer tissues and their corresponding adjacent normal tissues were stained with FAM3C, YY1 and HSF1 antibodies, respectively. For immunohistochemistry, sections were incubated with 3% hydrogen peroxide to block endogenous peroxidase activity. Tissue sections were blocked with 10% BSA for 1 hour and incubated with primary antibodies at 4°C overnight. A 1:100 dilution of anti‐FAM3C or anti‐YY1 or anti‐HSF1 antibody was used as the primary antibody. The use of human breast cancer tissues was approved by Medical‐ethical Committee of Peking University Health Science Center and China‐Japan Friendship Hospital (Permit Number ZRLW‐5). This study complies with the current laws in China.

### 2.12 Statistical analysis

The results are presented as the mean ± SEM. Statistical significance of differences between groups were analysed by *t* test. *P* values <0.05 were considered as statistically significant.

## 3 RESULTS

### 3.1 FAM3C promoted the proliferation and migration of MDA‐MB‐231 cells concomitant with HSF1 up‐regulation

**Figure 1:**
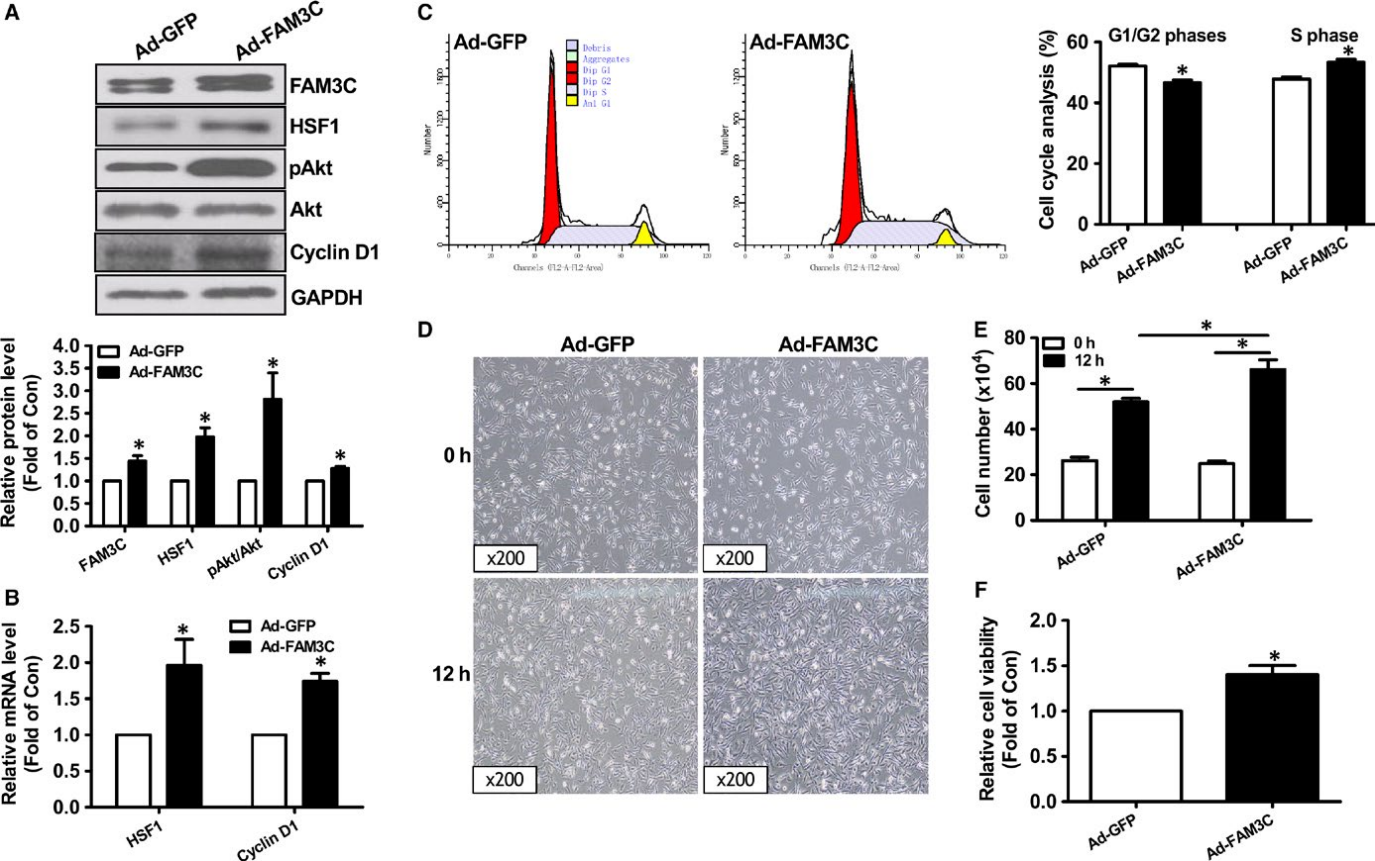
FAM3C overexpression induced HSF1 expression and promoted proliferation of MDA‐MB‐231 cells. A, FAM3C overexpression on the protein levels of HSF1, pAkt and Cyclin D1. Representative gel images were shown in upper panel, and quantitative data shown in lower panel. B, FAM3C overexpression on the mRNA levels of HSF1 and Cyclin D1. C, Cell cycle analyses after FAM3C overexpression. Representative analytic data shown in left panel, and quantitative data shown in right panel. D, Representative images of cell density after FAM3C overexpression. The amplification power had been marked in the images. E, Cell number counting assays after FAM3C overexpression. F, Cell viability assays after FAM3C overexpression. N = 3‐5, *P < 0.05 vs Ad‐GFP–treated cells or between two indicated group of cells. FAM3C, family with sequence similarity three member C; HSF1, heat shock factor 1; Akt, protein kinase B

**Figure 2:**
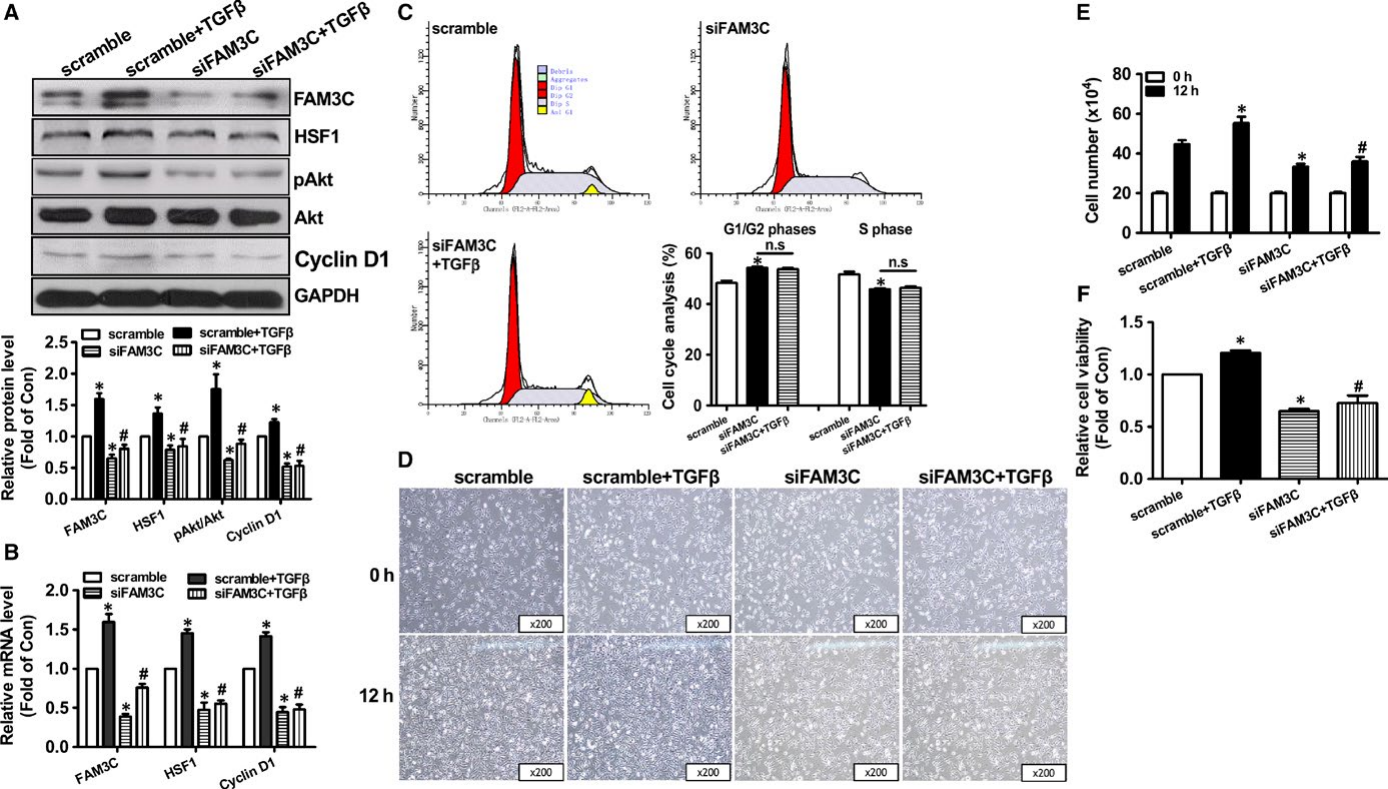
FAM3C silencing reduced HSF1 expression and inhibited TGFβ‐induced proliferation of MDA‐MB‐231 cells. A, FAM3C silencing on the protein levels of HSF1, pAkt and Cyclin D1. Representative gel images were shown in upper panel, and quantitative data shown in lower panel. B, FAM3C silencing on the mRNA levels of HSF1 and Cyclin D1. C, Cell cycle analyses after FAM3C silencing. D, Representative images of cell density after FAM3C silencing. E, Cell number counting assays after FAM3C silencing. F, Cell viability assays after FAM3C silencing. N = 3‐6, *P < 0.05 vs scramble‐treated cells, #*P* < 0.05 vs scramble + TGFβ group of cells. FAM3C, family with sequence similarity three member C; HSF1, heat shock factor 1; TGFβ, transforming growth factor beta; Akt, protein kinase B

Because TGFβ activation is highly associated with breast cancer, it has been chosen to establish a proliferation model of human breast cancer MDA‐MB‐231 cells (Figure S1A). TGFβ treatment up‐regulated the mRNA and protein levels of FAM3C and HSF1 by about 2–3‐fold in MDA‐MB‐231 cells (Figure S1B‐D). Generally, two FAM3C protein isoforms were detected in breast cancer cells and other cell types. The bigger one is the full length isoform (26kD), and the smaller one is the isoform after the cleavage of signalling peptide (22kD).^27^, ^28^, ^32^ In this study, the expressional levels of both FAM3C protein isoforms were quantitated together. To directly evaluate the impact of FAM3C on HSF1 expression, and proliferation and migration of breast cancer cells, FAM3C was overexpressed or inhibited in MDA‐MB‐231 cells. FAM3C overexpression by about 1.5‐fold significantly up‐regulated the protein levels of HSF1, pAkt and Cyclin D1, and the mRNA levels of HSF1 and Cyclin D1 by about 2–3‐fold when compared with Ad‐GFP–treated cells (Figure 1A,B). FAM3C overexpression increased the cells in S phase (Ad‐GFP 47.9 ± 0.4% vs 53.4 ± 0.8% Ad‐FAM3C, *P* < 0.05) and decreased the cells in G1/G2 phases (Ad‐GFP 52.1 ± 0.5% vs 46.6 ± 0.8 Ad‐FAM3C, *P* < 0.05) (Figure 1C). Furthermore, FAM3C overexpression promoted the proliferation of breast cancer cells as evaluated by morphological observation, cell number counting and cell viability assays, respectively (Figure 1D‐F), which supported the changes in proliferatic genes and cell cycle after FAM3C overexpression. FAM3C overexpression enhanced the migration of MDA‐MB‐231 cells (Figure S2A,B). siRNA transfection inhibited FAM3C protein expression by about 40% (Figure 2A). siRNA‐mediated FAM3C silencing reduced the mRNA and protein levels of HSF1 and Cyclin D1, and Akt phosphorylation by about 30%–50% in MDA‐MB‐231 cells (Figure 2A,B). Notably, FAM3C silencing inhibited TGFβ‐induced increase in HSF1, pAkt and Cyclin D1 expression levels (Figure 2A,B). FAM3C silencing reduced the cells in S phase (51.8 ± 0.7%, 45.8 ± 0.1% and 46.3 ± 0.4 for scramble, siFAM3C and siFAM3C+TGFβ group of cells, respectively; *P* < 0.05 between scramble and siFAM3C groups, there was no difference between siFAM3C and siFAM3C+TGFβ groups) and increased the cells in G1/G2 phases (48.2 ± 0.8%, 54.2 ± 0.3% and 53.7 ± 0.5% for scramble, siFAM3C and siFAM3C + TGFβ group of cells, respectively; *P* < 0.05 between scramble and siFAM3C groups, there was no difference between siFAM3C and siFAM3C+TGFβ groups), and TGFβ failed to enhance cell division after FAM3C silencing (Figure 2C). FAM3C silencing inhibited the proliferation of breast cancer cells in the absence or presence of TGFβ, which were consistent with the changes in cell cycle and proliferatic proteins (Figure 2D‐F). Because wound area calculation is more sensitive and accurate than migration distance measurement in evaluating cell migration, it had been used in further experiments. FAM3C silencing inhibited the migration of breast cancer cells in the absence or presence of TGFβ stimulation (Figure S3).

### 3.2 Inhibition of HSF1 reversed the stimulatory effects OF FAM3C and TGFβ on the proliferation and migration of MDA‐MB‐231 cells

**Figure 3:**
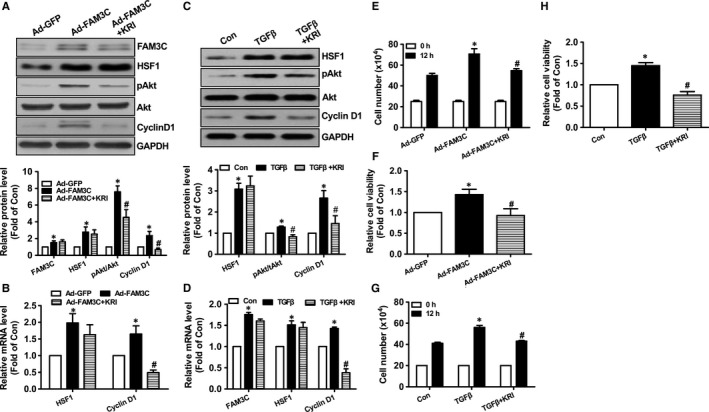
Inhibition of HSF1 repressed FAM3C and TGFβ‐induced proliferation of MDA‐MB‐231 cells. A, Inhibition of HSF1 repressed FAM3C‐induced Akt activation and Cyclin D1 up‐regulation. Representative gel images were shown in upper panel, and quantitative data shown in lower panel. B, Inhibition of HSF1 on FAM3C‐induced change in HSF1 and Cyclin D1 mRNA levels. C, Inhibition of HSF1 repressed TGFβ‐induced Akt activation and Cyclin D1 up‐regulation. Representative gel images shown in upper panel, and quantitative data shown in lower panel. D, Inhibition of HSF1 on TGFβ‐induced change in HSF1 and Cyclin D1 mRNA levels. E, Cell number counting assays after HSF1 inhibition in Ad‐FAM3C‐treated cells. F, Cell viability assays after HSF1 inhibition in Ad‐FAM3C–treated cells. G, Cell number counting assays after HSF1 inhibition in TGFβ‐treated cells. H, Cell viability assays after HSF1 inhibition in TGFβ‐treated cells. KRI, Ad‐FAM3C–infected or TGFβ‐treated cells in the presence of HSF1 inhibitor KRIBB11. N = 3‐4, *P < 0.05 vs Ad‐GFP–treated or control cells, #*P* < 0.05 vs Ad‐FAM3C‐infected or TGFβ‐treated cells. FAM3C, family with sequence similarity three member C; HSF1, heat shock factor 1; TGFβ, transforming growth factor beta; Akt, protein kinase B; KRI, KRIBB11, a inhibitor of HSF1

**Figure 4:**
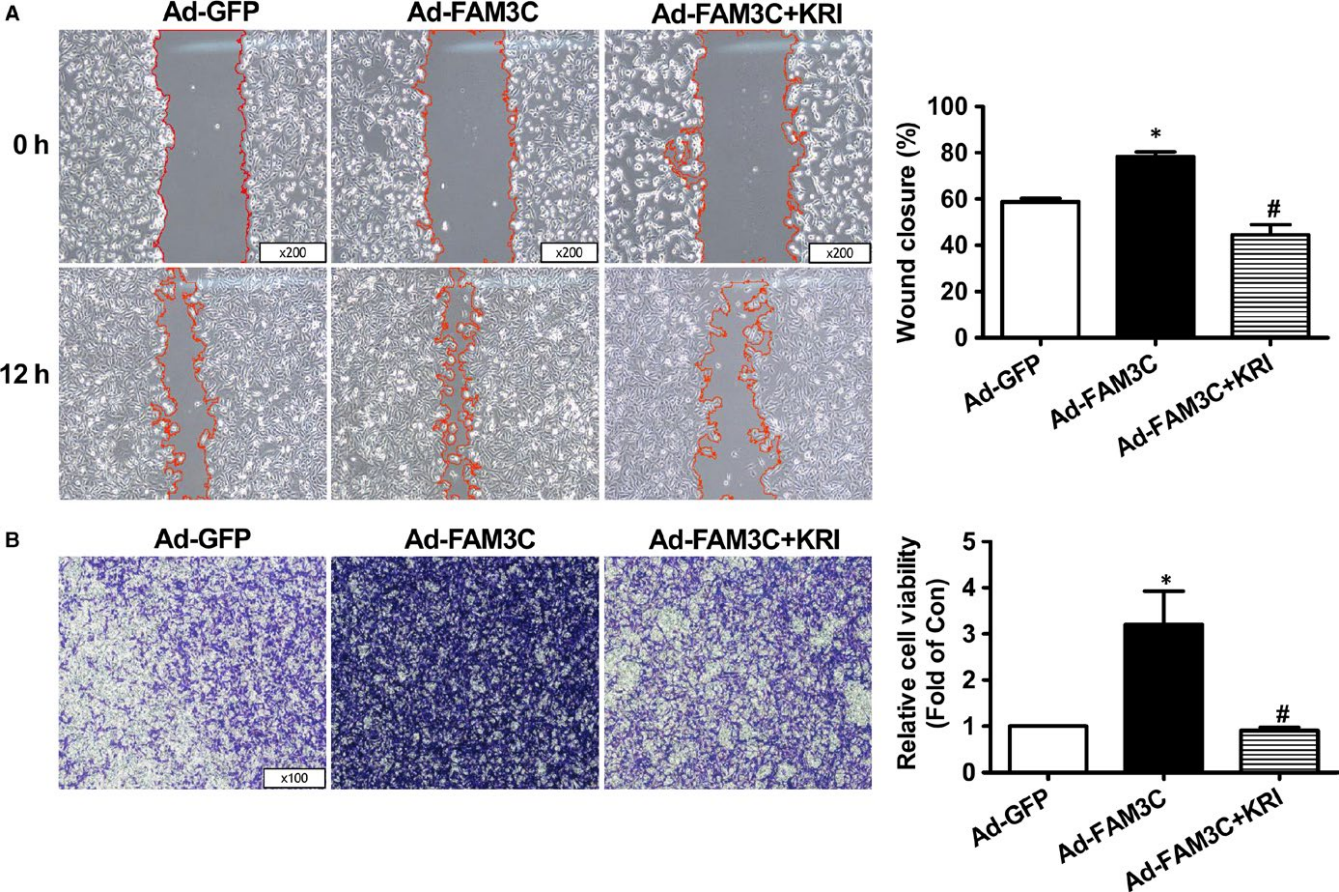
Inhibition of HSF1 repressed FAM3C‐induced migration of MDA‐MB‐231 cells. A, Inhibition of HSF1 repressed FAM3C‐induced migration of cells as evaluated by wound area calculation. Representative images were shown in left panel, and quantitative data shown in right panel. B, Inhibition of HSF1 repressed FAM3C‐induced migration of cells as evaluated by transwell migration assays. Representative images were shown in left panel, and quantitative data shown in right panel. KRI, Ad‐FAM3C–infected cells treated with HSF1 inhibitor KRIBB11. N = 3‐4, *P < 0.05 vs Ad‐GFP group of cells, #*P* < 0.05 vs Ad‐FAM3C–treated cells. FAM3C, family with sequence similarity three member C; HSF1, heat shock factor 1; KRI, KRIBB11, a inhibitor of HSF1

To further determine the roles of HSF1 in FAM3C‐ and TGFβ‐promoted proliferation and migration of breast cancer cells, its activity was blocked using a selective inhibitor KRIBB11. Treatment with KRIBB11 repressed FAM3C‐ and TGFβ‐induced Akt activation and Cyclin D1 expression with little effect on FAM3C and HSF1 expression (Figure 3A‐D). Cell number counting and cell viability assays revealed that inhibition of HSF1 abolished the stimulatory effects of FAM3C and TGFβ on the proliferation of MDA‐MB‐231 cells (Figure 3E‐H). Moreover, HSF1 inhibitor treatment had little effect on FAM3C expression (Figure 3A & D). HSF1 inhibition abolished the stimulatory effects of FAM3C on the migration of breast cancer cells as evaluated by both wound area calculation and transwell migration assays (Figure 4A‐B). Inhibition of HSF1 also blocked TGFβ‐induced migration of breast cancer cells (Figure S4). Plasmid overexpression of HSF1 induced Akt phosphorylation, which was inhibited by KRIBB11 in MDA‐MB‐231 cells (Figure S5A). HSF1 overexpression also stimulated the proliferation of MDA‐MB‐231 cells, but was reversed by KRIBB11 (Figure S5B‐D). Overall, these findings revealed that FAM3C stimulated the proliferation and migration of breast cancer cells by activating HSF1‐Akt pathway. Furthermore, TGFβ‐induced proliferation and migration of breast cancer cells is dependent on FAM3C‐HSF1‐Akt pathway in MDA‐MB‐231 cells.

### 3.3 YY1 activated HSF1 to induce the proliferation of breast cancer cells

**Figure 5:**
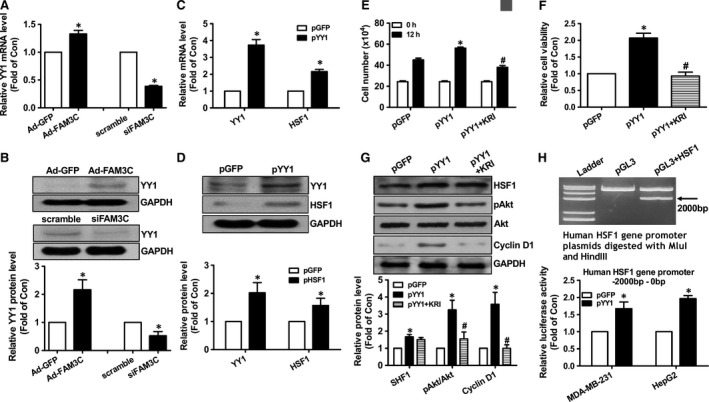
YY1 activated HSF1 expression to promote the proliferation of MDA‐MB‐231 cells. (A,B) FAM3C overexpression or silencing on the mRNA (A) and protein levels (B) of YY1 in MDA‐MB‐231 cells. (C,D) Plasmid overexpression of YY1 increased the mRNA (C) and protein levels (D) of HSF1 in MDA‐MB‐231 cells. (E,F) HSF1 inhibition repressed YY1‐induced proliferation of MDA‐MB‐231 cells. Cell counting data were shown in panel E, and cell viability analytic data shown in panel F. G, HSF1 inhibition repressed YY1‐induced Akt activation in MDA‐MB‐231 cells. H, YY1 activated the promoter activity of human HSF1 gene in MDA‐MB‐231 cells and HepG2 cells. KRI, pYY1‐transfected cells treated with HSF1 inhibitor KRIBB11. N = 3‐5, *P < 0.05 vs control cells, #*P* < 0.05 vs pYY1‐transfected cells. FAM3C, family with sequence similarity three member C; YY1, Ying‐Yang 1; HSF1, heat shock factor 1; Akt, protein kinase B; KRI, KRIBB11, a inhibitor of HSF1

Because FAM3C overexpression or silencing is associated with HSF1 mRNA level change in breast cancer cells, it is reasonable to speculate that FAM3C enhanced HSF1 gene transcription. To identify the potential transcription factors that mediate FAM3C‐induced transcription enhancement of HSF1 gene, the putative binding sites for some certain important transcription factors in human HSF1 gene promoter between −2000 bp and the transcription start site (0 bp) had been analysed using bioinformatic method. The potential binding sites with high prediction scores for certain transcription factors were indicated in Figure S6. As a result, bioinformatic prediction revealed that six putative binding sites highly specific for transcription factor YY1 were existed in the promoter region of human HSF1 gene (Figure S6). Reference mining revealed that YY1 deregulation had been reported to be involved in the pathogenesis of breast cancer.^33^, ^34^ Thus, whether FAM3C enhanced HSF1 transcription through YY1 had been determined. FAM3C overexpression increased the mRNA and protein levels of YY1, whereas FAM3C silencing reduced them in human MDA‐MB‐231 cells (Figure 5A,B). Plasmid overexpression of YY1 significantly up‐regulated the mRNA and protein levels of HSF1 in MDA‐MB‐231 cells about 2‐fold (Figure 5C‐D). YY1 overexpression stimulated the proliferation of breast cancer cells (Figure 5E‐F), but was blocked by HSF1 inhibitor. HSF1 inhibition also repressed YY1‐induced migration of breast cancer cells (Figure S7). YY1 overexpression induced Akt activation and up‐regulated Cyclin D1, but were blocked by HSF1 inhibitor (Figure 5G). To further confirm whether YY1 directly activated the transcription of HSF1 gene, a human HSF1 gene promoter fragment flanking −2000 to 0 bp had been cloned. Luciferase reporter assays indicated that YY1 activated HSF1 gene promoter activity in both human MDA‐MB‐231 cells and HepG2 cells by about 1.5–2‐fold (Figure 5H). Overall, these findings revealed that HSF1 is a direct target gene of YY1, which stimulated the proliferation and migration of breast cancer cells by inducing HSF1 expression.

### 3.4 FAM3C and TGFβ promoted the proliferation and migration of breast cancer cells via YY1‐HSF1 pathway

**Figure 6:**
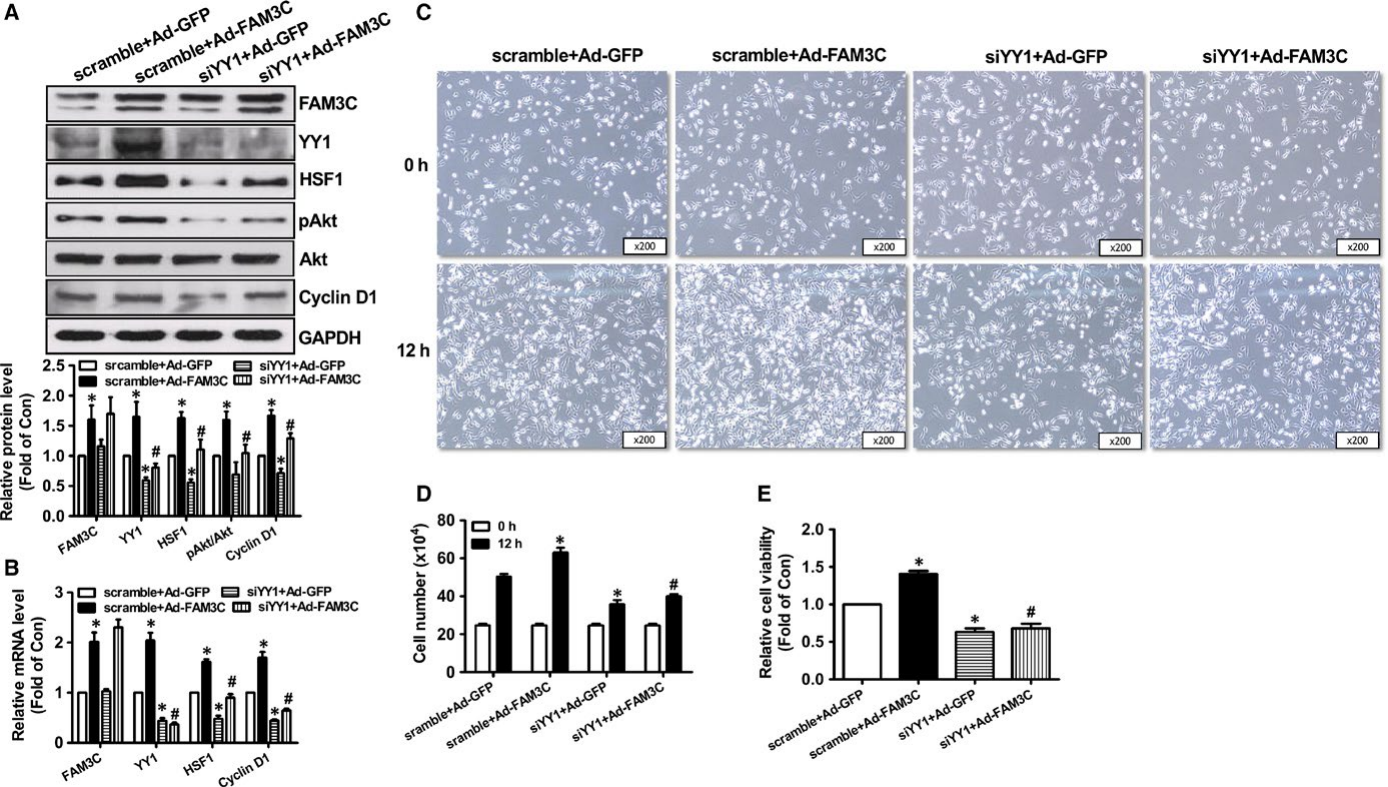
Silencing of YY1 repressed FAM3C‐induced HSF1 up‐regulation and proliferation of MDA‐MB‐231 cells. A, Silencing of YY1 repressed FAM3C‐induced increase in HSF1 protein and Akt activation. Representative gel images were shown in upper panel, and quantitative data shown in lower panel. B, Silencing of YY1 repressed FAM3C‐induced increase in HSF1 and Cyclin D1 mRNA levels. C, Representative images of cell density after YY1 inhibition. D, Cell number counting assays after YY1 inhibition. E, Cell viability assays after YY1 inhibition. N = 3‐5, *P < 0.05 vs scramble + Ad‐GFP group of cells, #*P* < 0.05 vs scramble + Ad‐FAM3C group of cells. FAM3C, family with sequence similarity three member C; YY1, Ying‐Yang 1; HSF1, heat shock factor 1; Akt, protein kinase B

**Figure 7:**
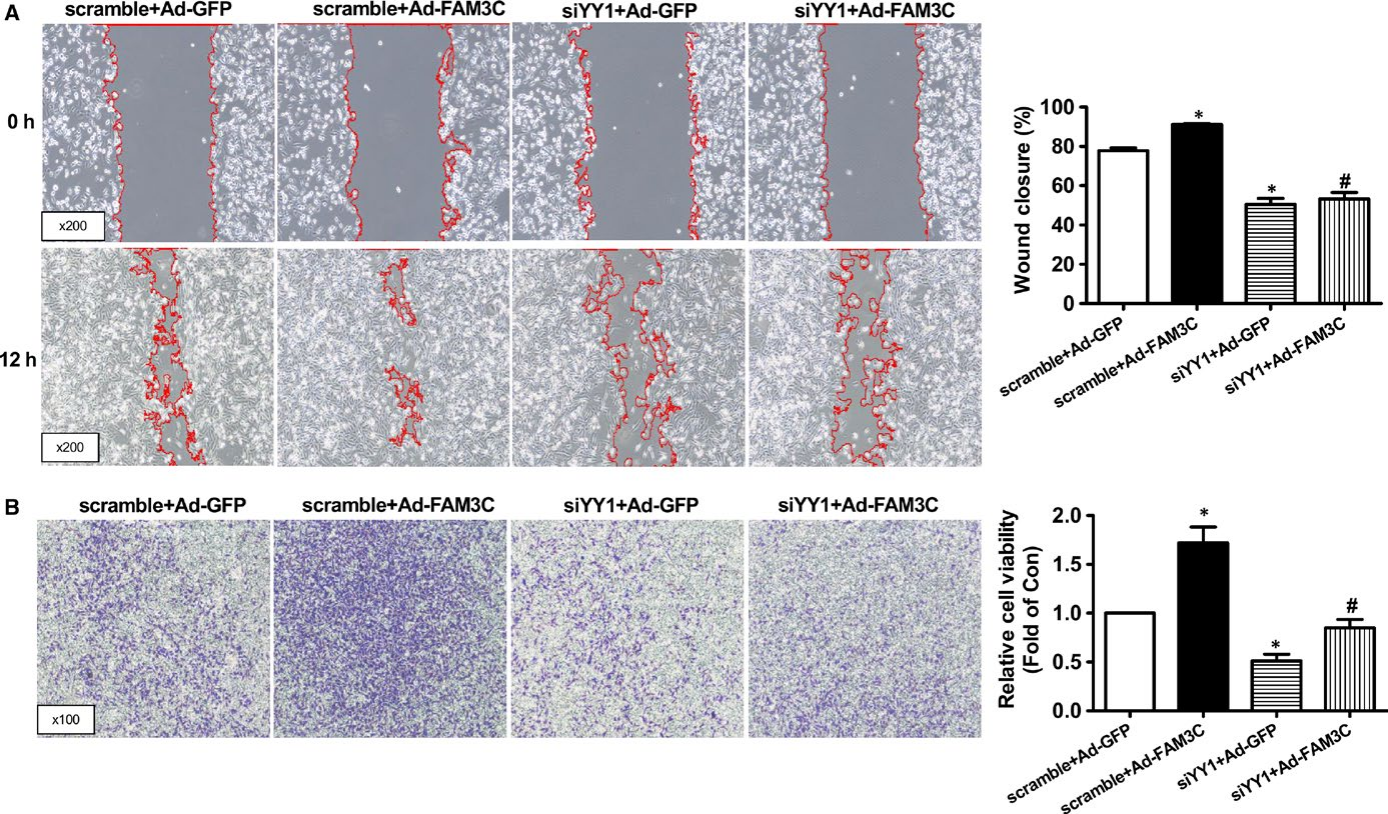
YY1 silencing inhibited FAM3C‐promoted migration of MDA‐MB‐231 cells. A, YY1 silencing inhibited FAM3C‐induced migration of breast cancer cells as evaluated by wound area calculation. Representative images were shown in left panel, and quantitative data shown in right panel. B, YY1 silencing inhibited FAM3C‐induced migration of breast cancer cells as evaluated by transwell migration assays. Representative images shown in left panel, and quantitative data shown in right panel. N = 3, *P < 0.05 vs scramble + Ad‐GFP group of cells, #*P* < 0.05 vs scramble + Ad‐FAM3C group of cells. FAM3C, family with sequence similarity three member C; YY1, Ying‐Yang 1

To further determine the role of YY1 in FAM3C‐induced HSF1 up‐regulation, and the proliferation and migration of breast cancer cells, YY1 expression was knockdown by siRNA transfection. siRNA transfection reduced the YY1 protein level by about 50% in MDA‐MB‐231 cells (Figure 6A). The mRNA and protein levels of HSF1 and Cyclin D1, and Akt phosphorylation were decreased by about 30%–60%, respectively, after YY1 silencing in MDA‐MB‐231 cells (Figure 6A,B). In contrast, silencing of YY1 had little effect on FAM3C expression, suggesting that YY1 is a downstream molecule of FAM3C (Figure 6A,B). YY1 silencing repressed FAM3C‐induced up‐regulation of the mRNA and protein levels of HSF1 and Cyclin D1, and pAkt (Figure 6A,B). Consistent with the changes in proliferatic genes, morphological observation, cell number counting and cell viability assays indicated that YY1 silencing inhibited proliferation of MDA‐MB‐231 cells with or without FAM3C overexpression (Figure 6C‐E). YY1 silencing also inhibited the migration of MDA‐MB‐231 cells with or without FAM3C overexpression as evaluated by wound area calculation (Figure 7A). Transwell migration assay confirmed that YY1 silencing blunted FAM3C‐induced migration of MDA‐MB‐231 cells (Figure 7B). Similarly, YY1 silencing also inhibited TGFβ‐induced up‐regulation of HSF1 and Cyclin D1 expressions, and Akt activation (Figure S8A,B), and the proliferation and migration of MDA‐MB‐231 cells (Figures S8C‐E, S9). To further confirm that TGFβ activated FAM3C‐YY1‐HSF1 pathway to promote the proliferation and migration of breast cancer cells, some key findings observed in MDA‐MB‐231 cells had been validated in another human breast cancer cell line BT‐549 cells. In BT‐549 cells, TGFβ‐treated cells had higher expression levels of FAM3C, YY1 and HSF1 mRNAs and proteins with Akt phosphorylation (Figure S10A,B) than control cells. Similarly, Ad‐FAM3C infection also up‐regulated the expression levels of YY1 and HSF1 mRNAs and proteins with Akt activation when compared with Ad‐GFP–treated cells (Figure S10C,D). Morphological observation and cell number counting assays indicated that FAM3C overexpression significantly stimulated the proliferation of BT‐549 cells, but was completely blocked by inhibitor of HSF1 (Figure S11). Similarly, TGFβ‐promoted proliferation of BT‐549 cells was also blocked by HSF1 inhibition (Figure S12). Furthermore, FAM3C overexpression and TGFβ treatment also significantly stimulated the migration of BT‐549 cells, which was blunted by HSF1 inhibition (Figure S13A,B). Collectively, these findings in MDA‐MB‐231 and BT‐549 cell lines together suggested that FAM3C and TGFβ promoted the proliferation and migration of breast cancer cells via the activation of YY1‐HSF1 signalling axis.

### 3.5 FAM3C‐YY1‐HSF1 signalling axis is activated in human breast cancer tissues

**Figure 8:**
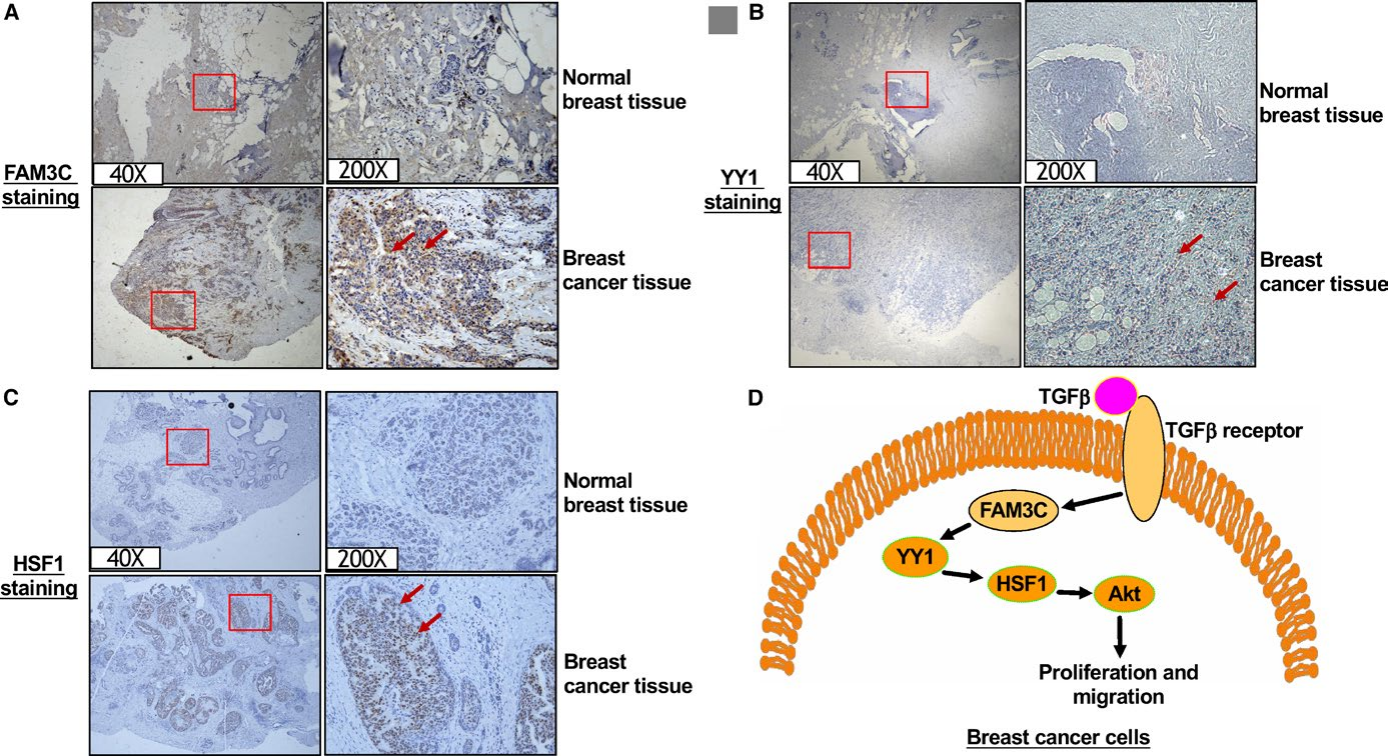
FAM3C‐YY1‐HSF1 axis was activated in human breast cancer tissues. (A‐C) Immunohistochemical staining revealed that FAM3C (A), YY1 (B) and HSF1 (C) protein levels were increased in breast cancer tissues when compared normal breast tissues. Three breast cancer tissues and their corresponding adjacent normal tissues were stained with FAM3C, YY1 and HSF1 antibodies, respectively. The amplification power had been marked in the images. The cells with positive staining by the indicated antibodies exhibited brown, and representative positive cells had been marked by arrows in the images. D, Proposed mode of FAM3C‐YY1‐HSF1 signalling axis in the pathogenesis of breast cancer. FAM3C‐YY1‐HSF1 signalling axis is essential for TGFβ‐promoted proliferation and migration of breast cancer cells. FAM3C, family with sequence similarity three member C; YY1, Ying‐Yang 1; HSF1, heat shock factor 1; TGFβ, transforming growth factor beta; Akt, protein kinase B

To validate the roles of FAM3C‐YY1‐HSF1 signalling axis in the pathogenesis of breast cancer, FAM3C, YY1 and HSF1 protein expressions were analysed in human breast cancer tissues. Immunohistochemical staining revealed the protein levels of FAM3C, YY1 and HSF1 were increased in human breast cancer tissues when compared with adjacent normal tissues (Figure 8A‐C, Figure S14A‐C).

## 4 DISCUSSION

The transcription factor YY1 was discovered in the 1990s 35, 36. YY1 belongs to the GLI‐Kruppel zinc finger transcription factor family, and has many biological functions by regulating the expressions of its target genes.^37^ It has been estimated that YY1 may regulate about 10% of mammalian gene expressions.^38^, ^39^ Recently, there had been increasing evidence that YY1 also plays important roles in the pathogenesis of various cancers including breast cancer.^40^, ^41^ In human breast cancer tissues, YY1 expression is increased, and high expression of YY1 protein level is associated with poor prognosis of breast cancer in human.^33^, ^34^, ^40^, ^41^ However, Lea MH et al also reported that YY1 protein level is lower in the breast cancer tissues of breast cancer patients than in the normal breast tissues.^33^ Moreover, YY1 overexpression represses the proliferation and migration of breast cancer cell lines.^33^ Clearly, more intensive studies are needed to clarify the role and mechanism of YY1 in breast cancer, in particular, at the different stage of tumour development.

FAM3C,^14^, ^15^ HSF1^23^, ^24^ and YY1^33^, ^34^ have been independently reported to be associated with breast cancer, and all of them could serve as potential diagnostic biomarkers and therapeutical targets. However, whether they are coordinated to promote the development of cancers remains unknown. This study revealed that HSF1 is a direct target gene of YY1, which induces HSF1 gene transcription. FAM3C up‐regulates YY1 to induce HSF1 expression, finally activating Akt‐Cyclin D1 pathway to promote the proliferation and migration of breast cancer cells. For the first time, our findings revealed that these important molecules are coordinated to promote the proliferation and migration of breast cancer cells. Clearly, the roles of FAM3C, YY1 and HSF1 in the pathogenesis of breast cancer and/or other cancers should be considered as a whole. The novel FAM3C‐YY1‐HSF1 signalling axis also provides a new explanation mechanism for TGFβ‐related breast cancer. An increase in FAM3C expression due to excessive TGFβ production plays important roles in inducing YY1 and HSF1 expressions as observed in human breast cancer or other cancer tissues. In one previous study, Chaudhury A et  al found that TGFβ‐induced EMT is associated with the up‐regulation of FAM3C and activation of Akt.^44^ However, the relationship between FAM3C up‐regulation and Akt activation remained unanswered in the same study.^44^ The current and our previous findings^27^, ^28^ revealed that FAM3C likely mediates TGFβ‐promoted Akt activation in the pathogenesis of EMT through YY1‐HSF1 signalling axis. New FAM3C‐YY1 pathway also provides an explanation for the findings that TGFβ induces HSF1 expression with unclear mechanism in ovarian and breast cancer cells.^45^, ^46^ Interestingly, although YY1‐HSF1 pathway plays an important role in activating Akt, it has also been reported that Akt activation also activates YY1 and HSF1 expressions.^47^, ^48^ TGFβ activated FAM3C‐YY1‐HSF1 axis to promote proliferation and migration in various breast cancer cell lines. Collectively, these findings together suggested that FAM3C likely initiates the crosstalks among YY1, HSF1 and Akt, finally causing excessive Akt activation to trigger tumour growth and invasion.

Given that Akt plays essential roles in regulating metabolism^7^ and promoting cancers,^49^ FAM3C‐YY1‐HSF1 signalling axis may play unique role in maintaining the balance between metabolism and cancers by modulating Akt activity. Under insulin resistance, repression of FAM3C by the factors such as fatty acids will impair YY1‐HSF1‐Akt pathway to enhance hepatic gluconeogenesis and lipogenesis, exaggerating hyperglycaemia and fatty liver.^27^, ^28^ However, in case of TGFβ overproduction, long‐term activation of FAM3C‐YY1‐HSF1 pathway will trigger the development of cancers in various tissues including breast tissue by promoting excessive Akt activation. Given the important roles of HSF1 in regulating hepatic glucose and lipid metabolism by modulating Akt activity and molecular chaperone expressions,^27^, ^28^, ^50^, ^51^ the side effects such as hyperglycaemia should be taken into consideration when HSF1 inhibitor is potentially used to treat cancers.

So far, the mechanism of FAM3C‐induced YY1 activation still needs further exploration. Moreover, because breast cancer cells had been previously shown to secrete FAM3C protein,^32^ it is of great significance to determine the association between circulating FAM3C protein and breast cancer progression in future. At present, it is difficult in accurately detecting the FAM3C protein level in the circulation due to the unavailability of high sensitive methods such as ELISA and RIA. Clearly, developing high sensitive methods for the determination of circulating FAM3C protein level will shed light on the diagnosis and treatment of breast cancer.

In summary, this study revealed that FAM3C, YY1 and HSF1 are coordinated to promote the proliferation and migration of human breast cancer MDA‐MB‐231 cells. FAM3C activates YY1 to induce HSF1 expression, which finally triggers the proliferation and migration of breast cancer cells by activating Akt. Furthermore, FAM3C‐YY1‐HSF1 signalling axis is essential for TGFβ‐promoted proliferation and migration of human breast cancer cells (Figure 8D). Targeting FAM3C‐YY1‐HSF1 pathway represents a potential strategy for treating TGFβ‐related breast cancer.

## CONFLICT OF INTEREST

The authors declare that they have no competing interests.

## AUTHORS' CONTRIBUTIONS

WY, BF and YM researched data and contributed to discussion. WY and JW provided the technical assistance. WY, BF and YM wrote the manuscript. BF, YM, BG, QC and JY revised/edited manuscript. HZ, YY and JY designed the study and revised the manuscript. HZ provided human tissue slides. Dr JY is the guarantor of this work, and have full access to all the data in the study and take responsibility for the integrity of the data and the accuracy of the data analysis. All authors read and approved the manuscript.
