# Supplementary material for: FAM3C‐YY1 axis is essential for TGFβ‐promoted proliferation and migration of human breast cancer MDA‐MB‐231 cells via the activation of HSF1
Source: J Cell Mol Med. 2019 Mar 19;23(5):3464–75. doi: 10.1111/jcmm.14243 (PMC6484506; doi:10.1111/jcmm.14243)

Suppl Figure 1

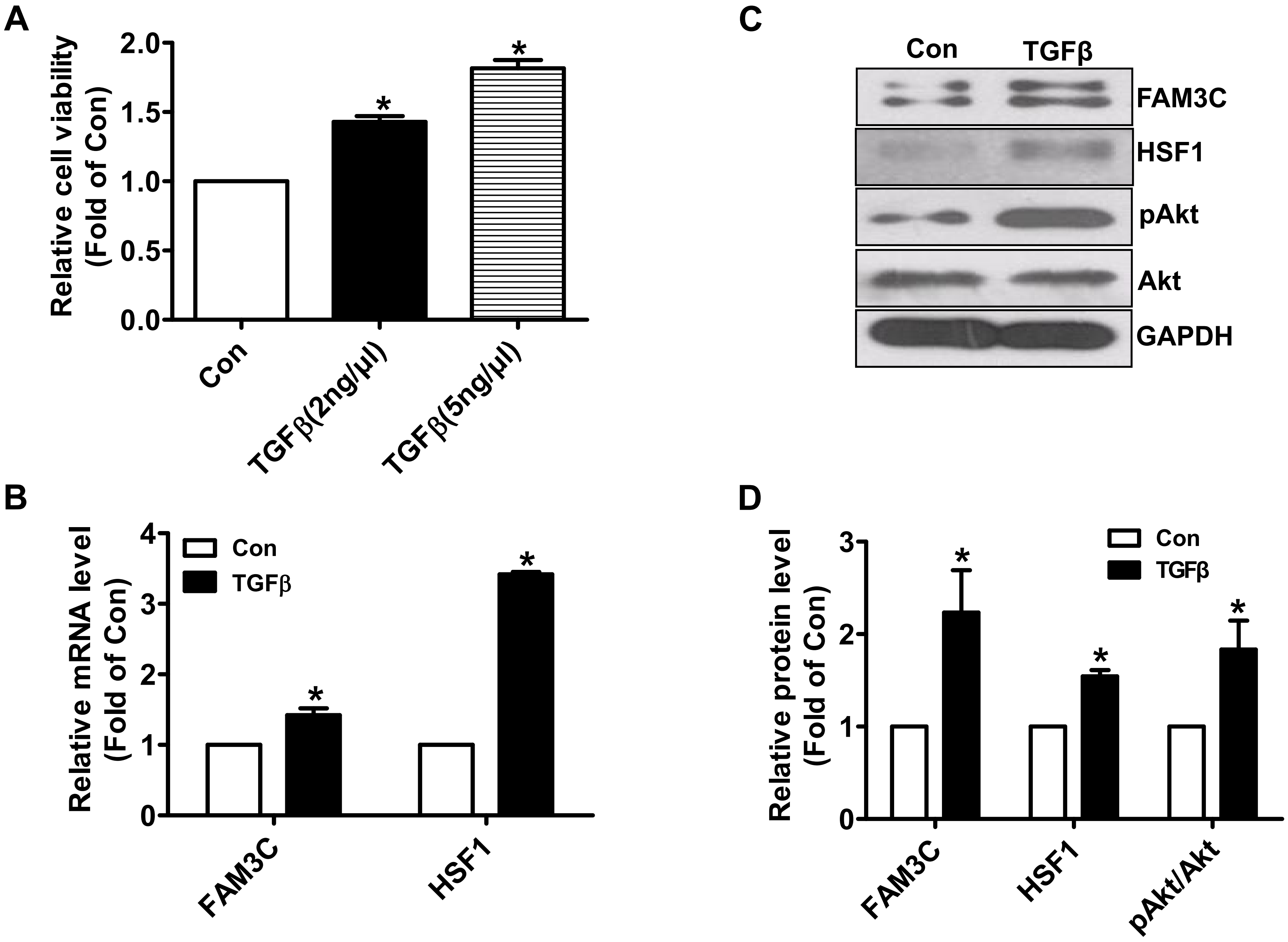

Suppl figure 2

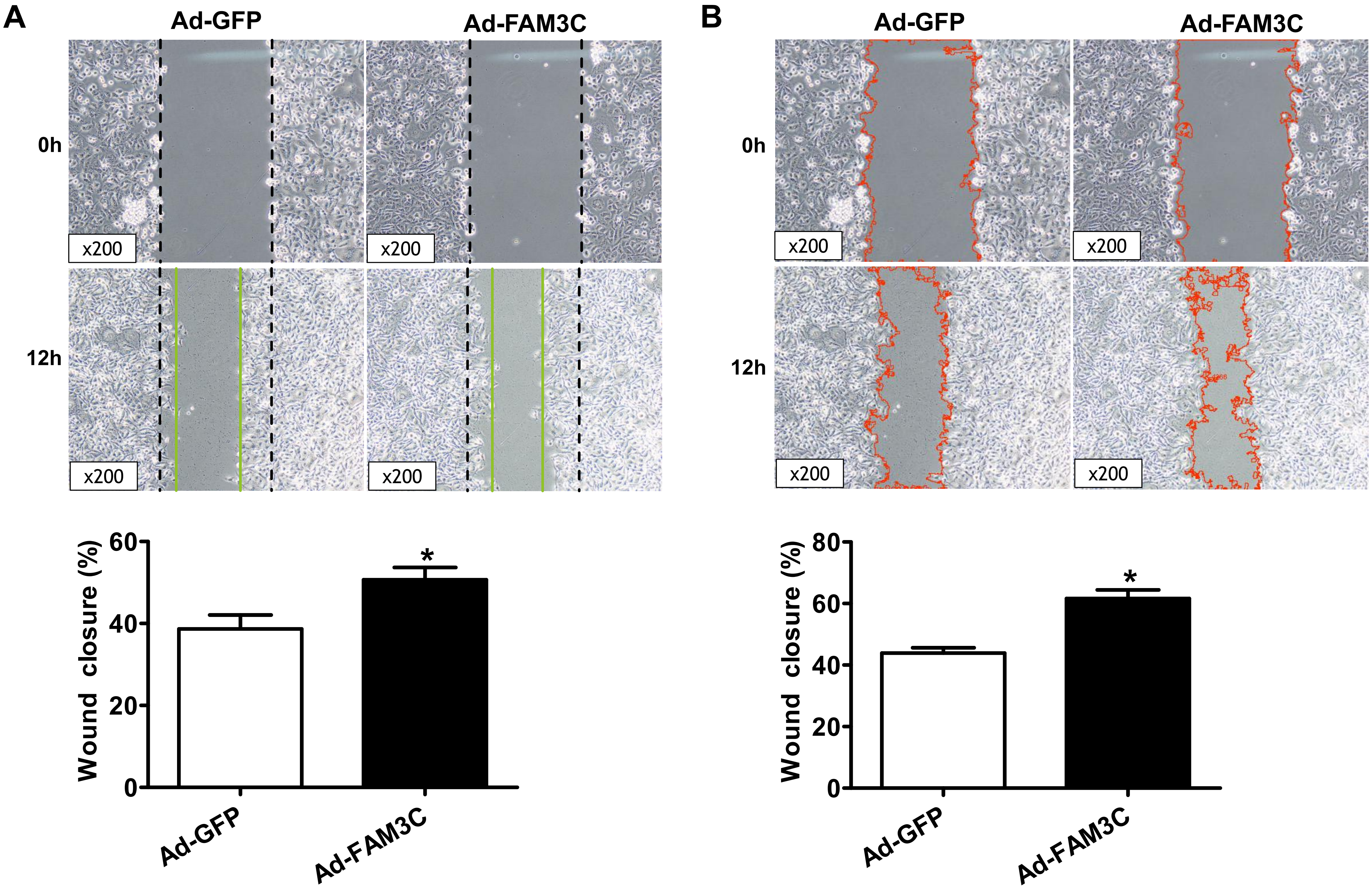

Suppl Figure 3

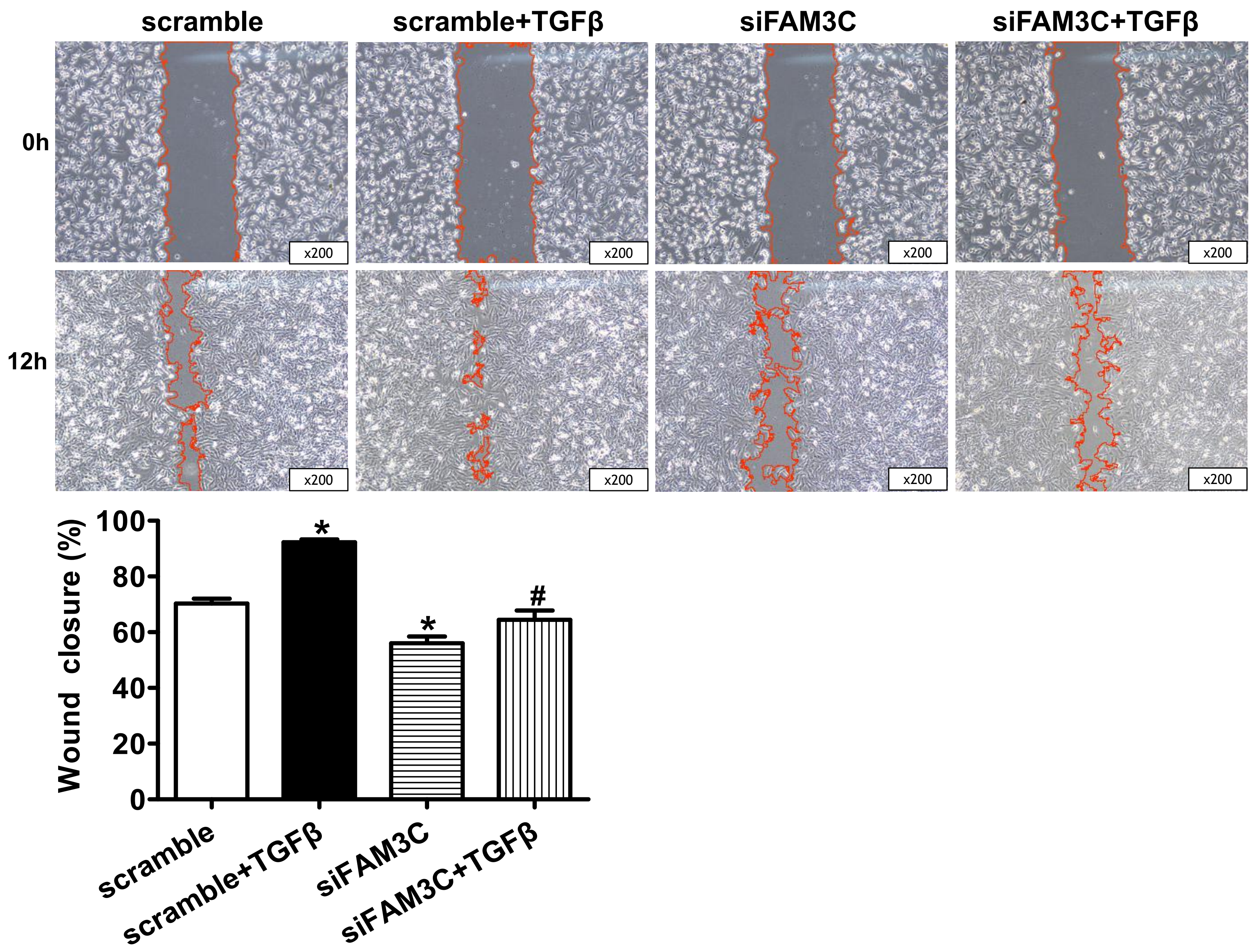

Suppl figure 4

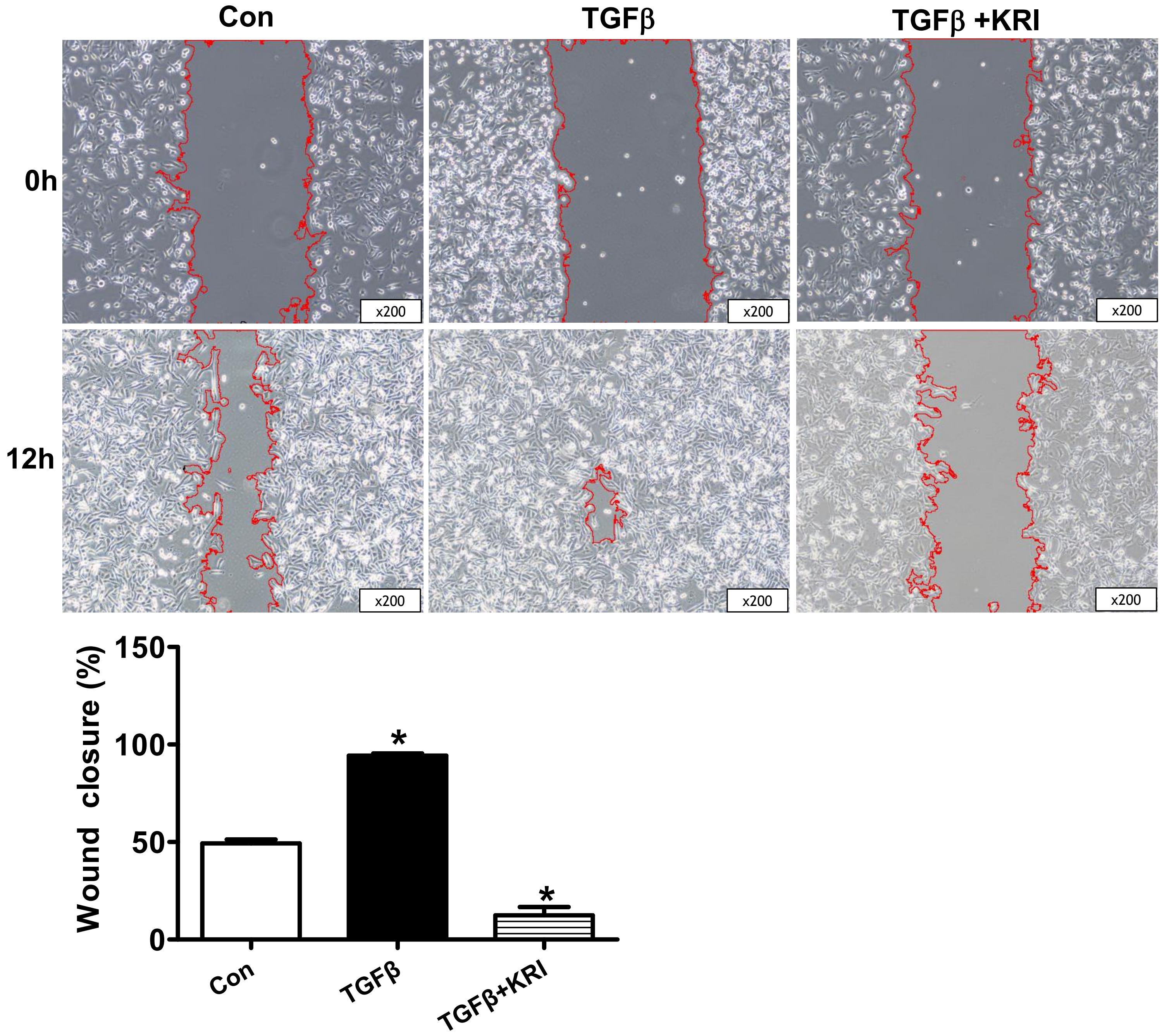

Suppl figure 5

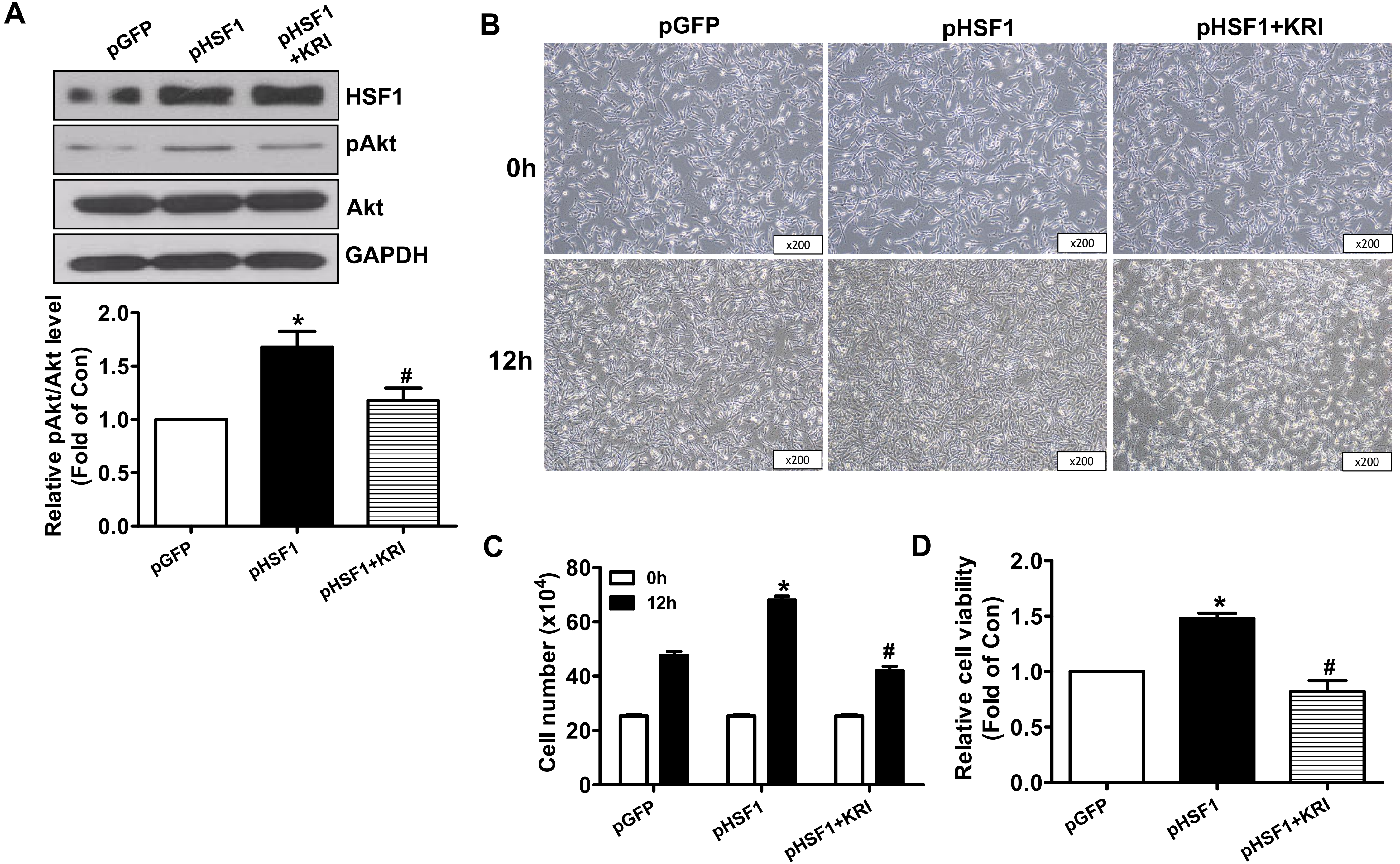

Suppl figure 6

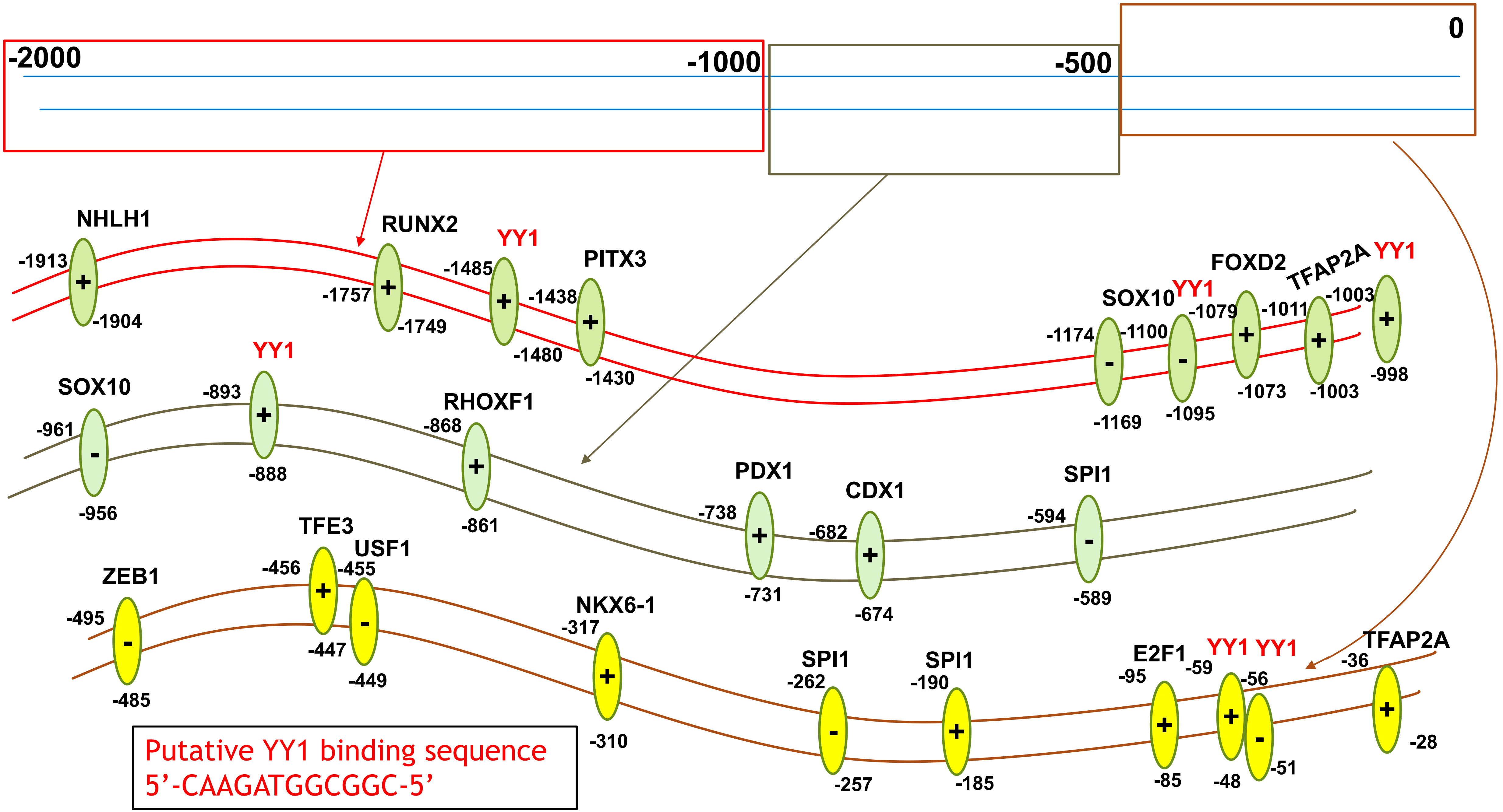

Suppl figure 7

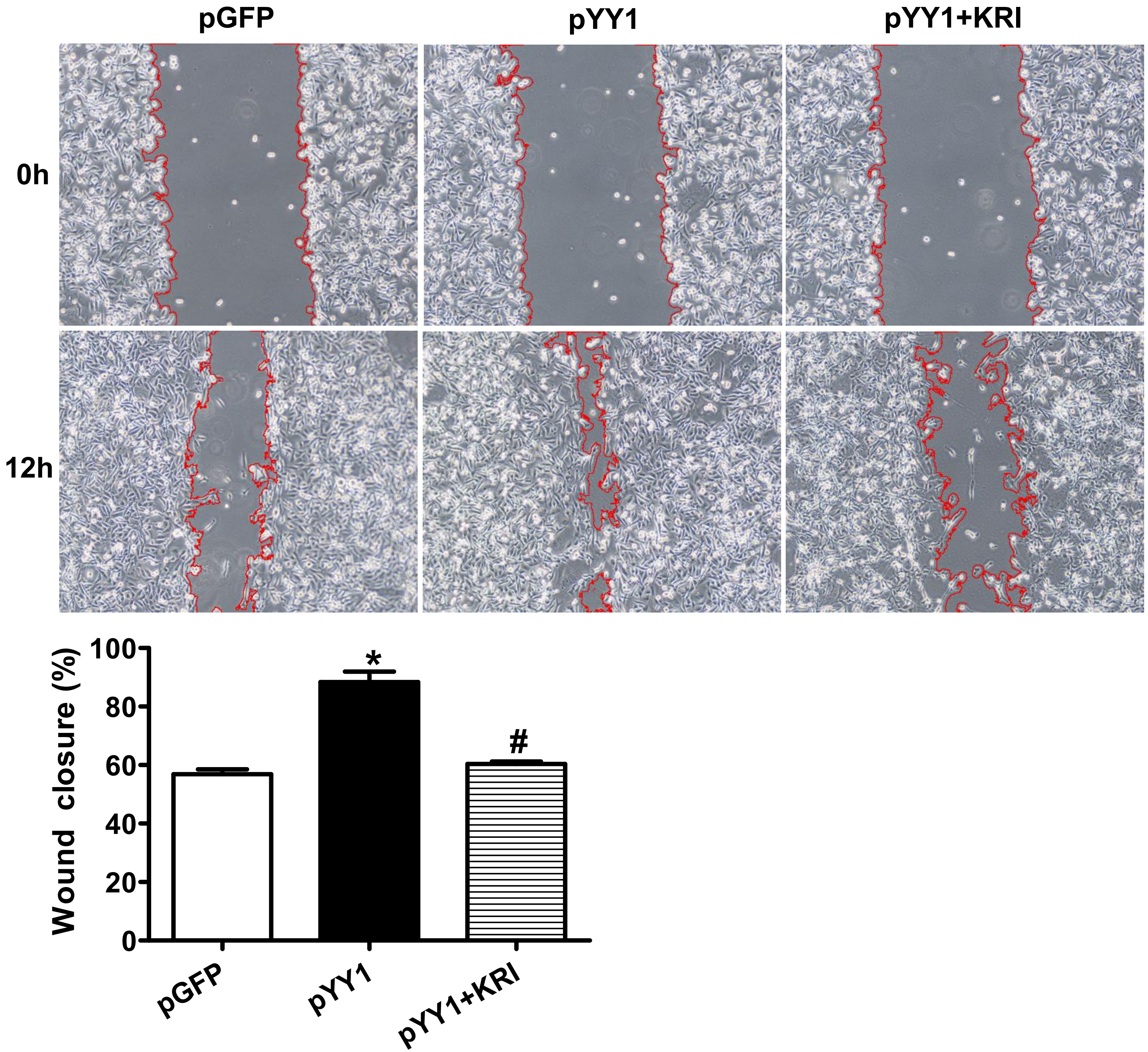

Suppl figure 8

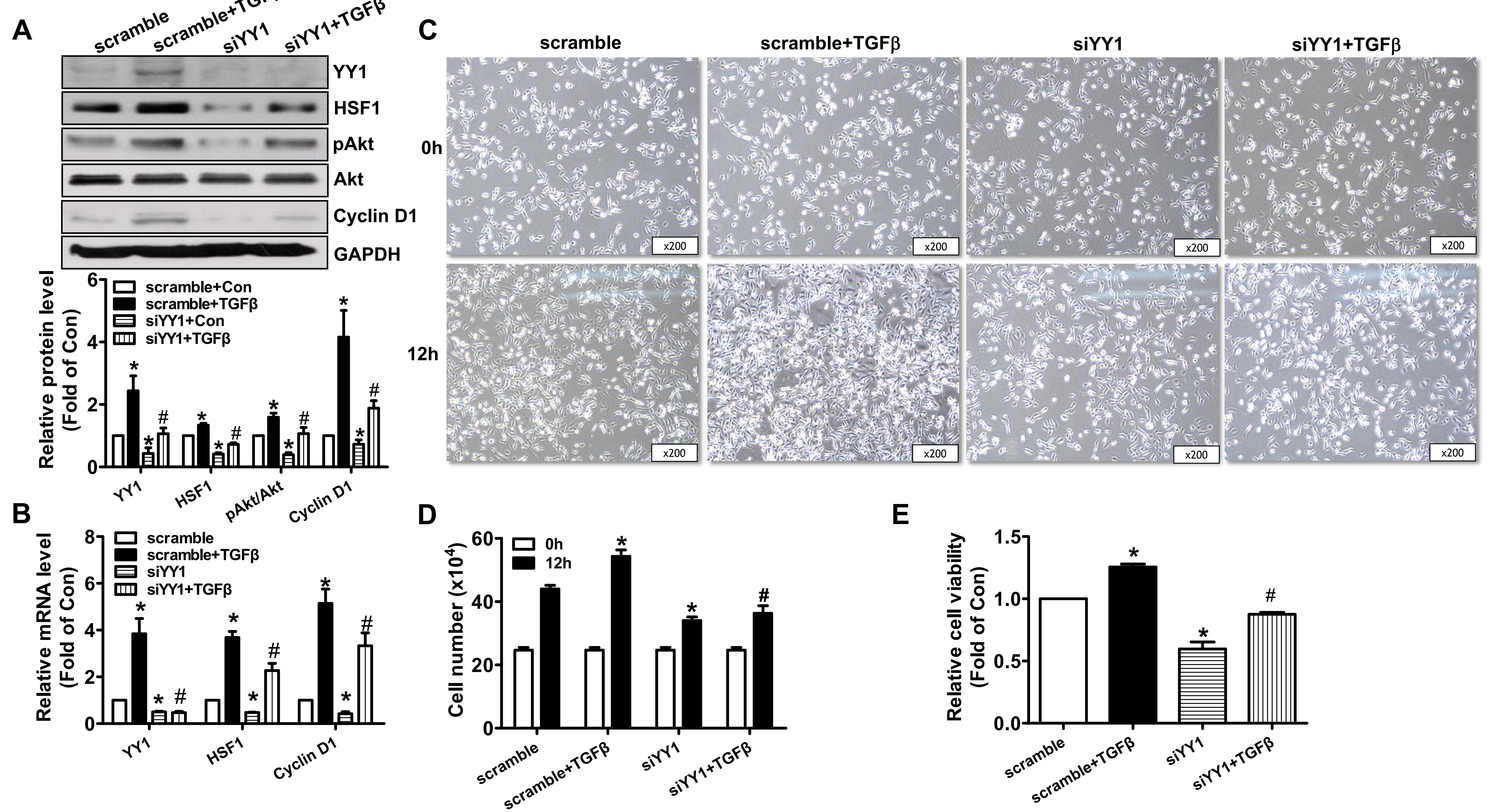

Suppl figure 9

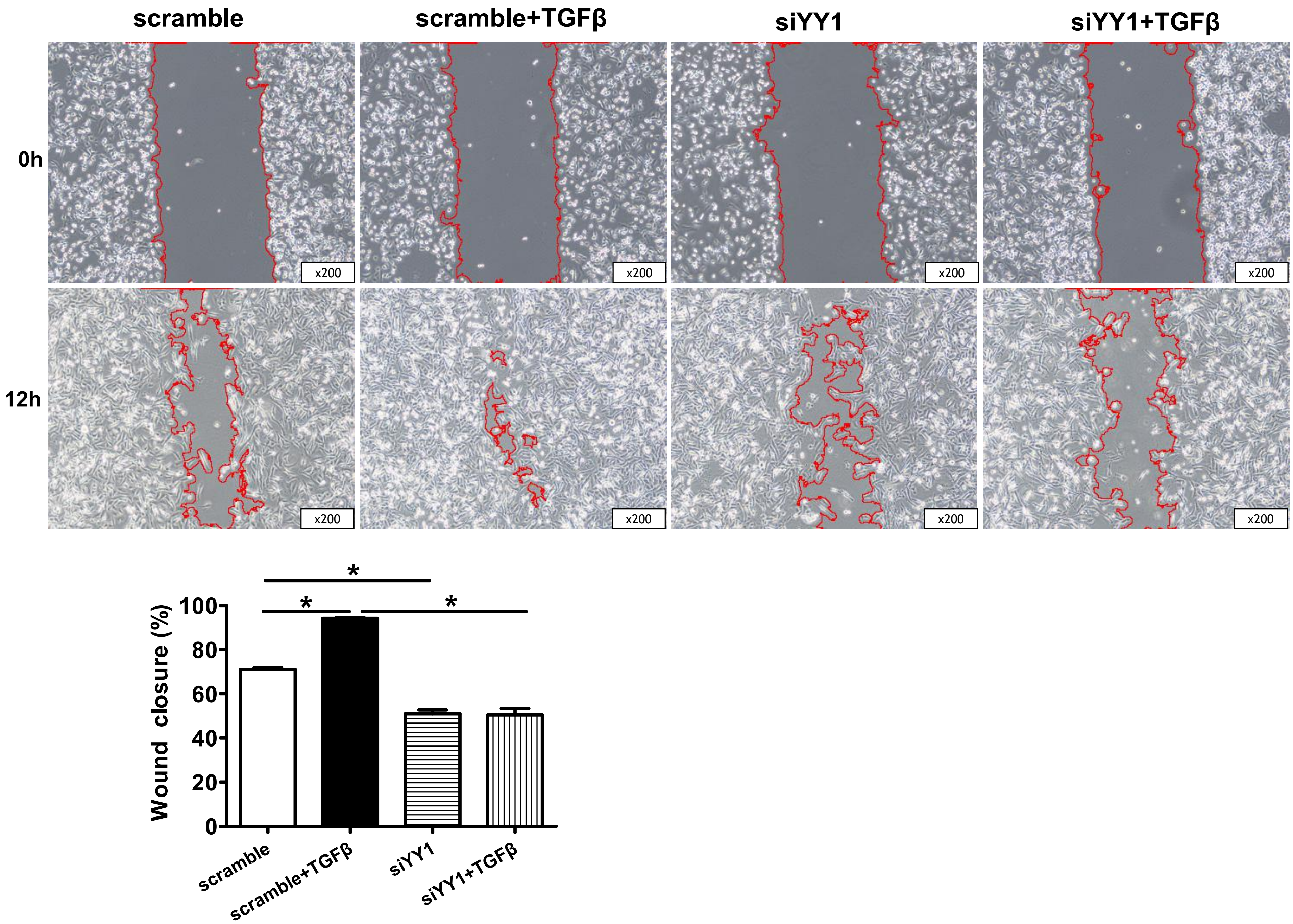

Suppl figure 10

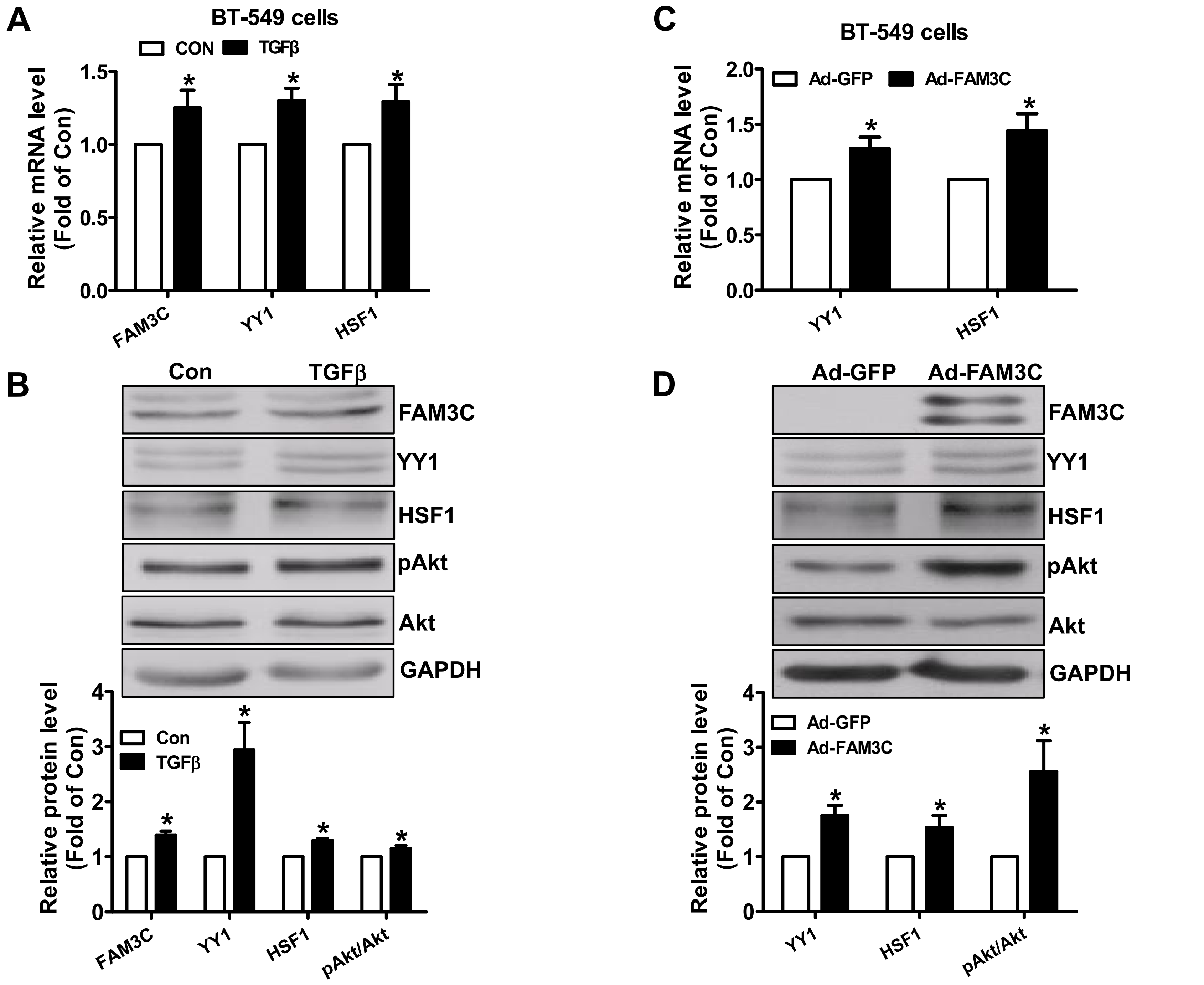

Suppl figure 11

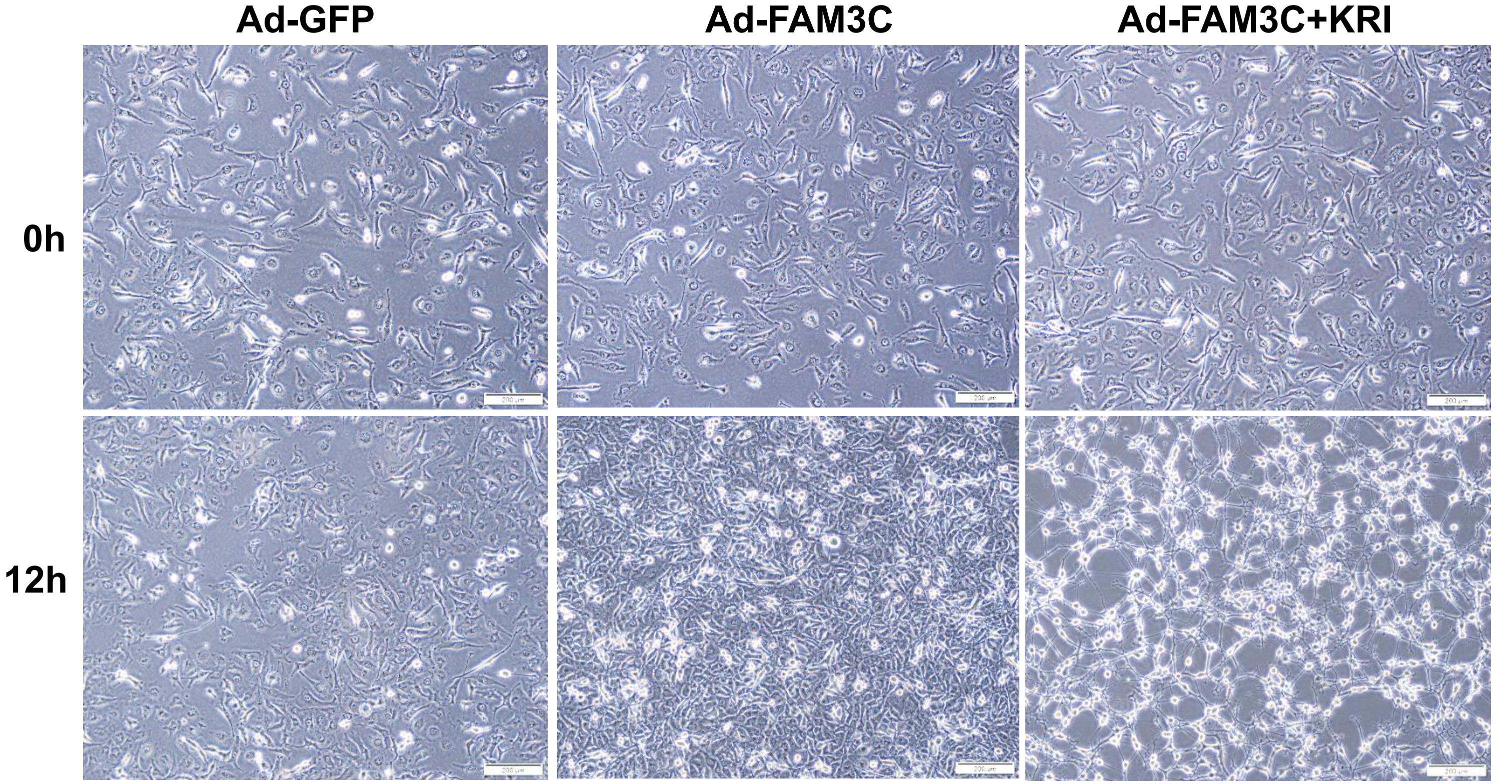

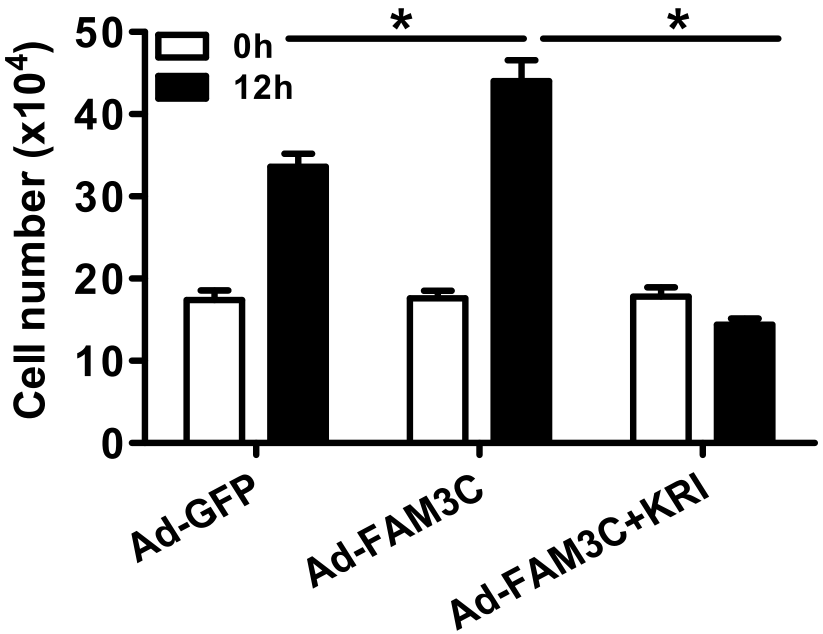

Suppl figure 12

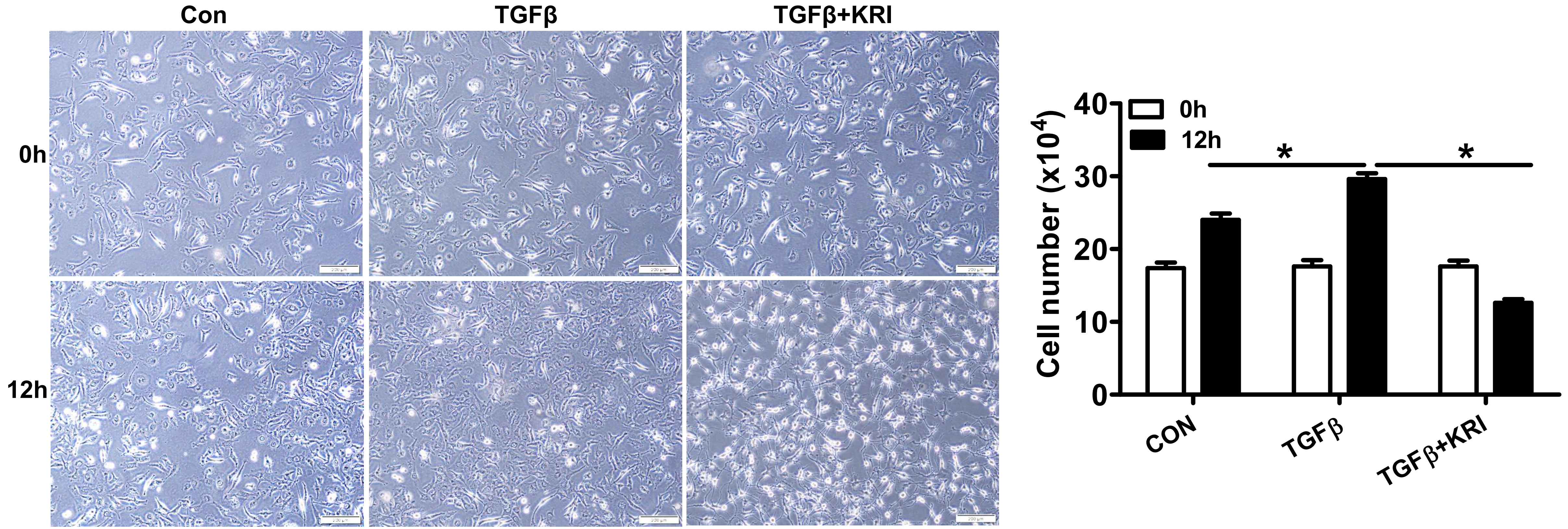

Suppl figure 13

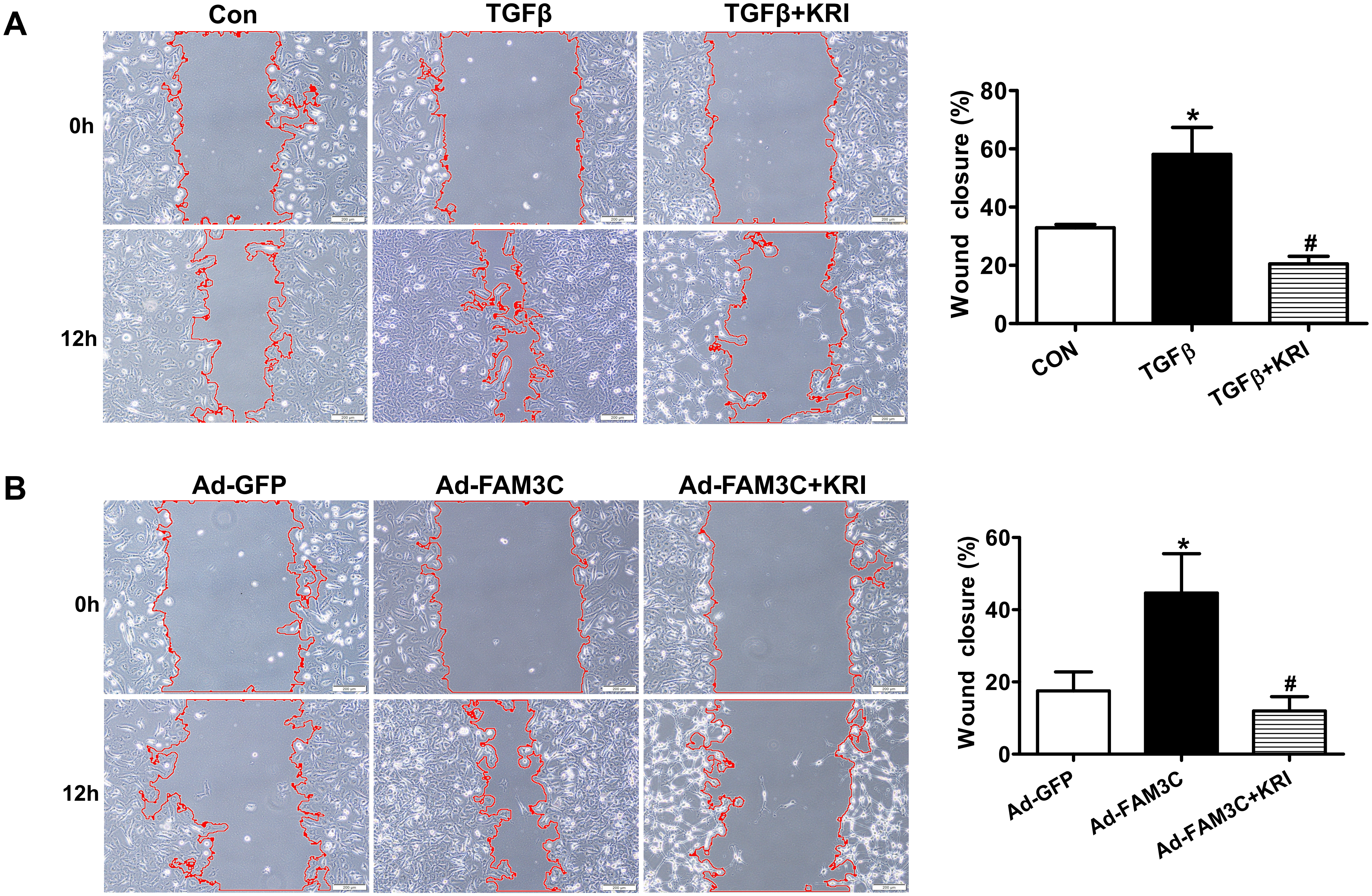

Suppl figure 14

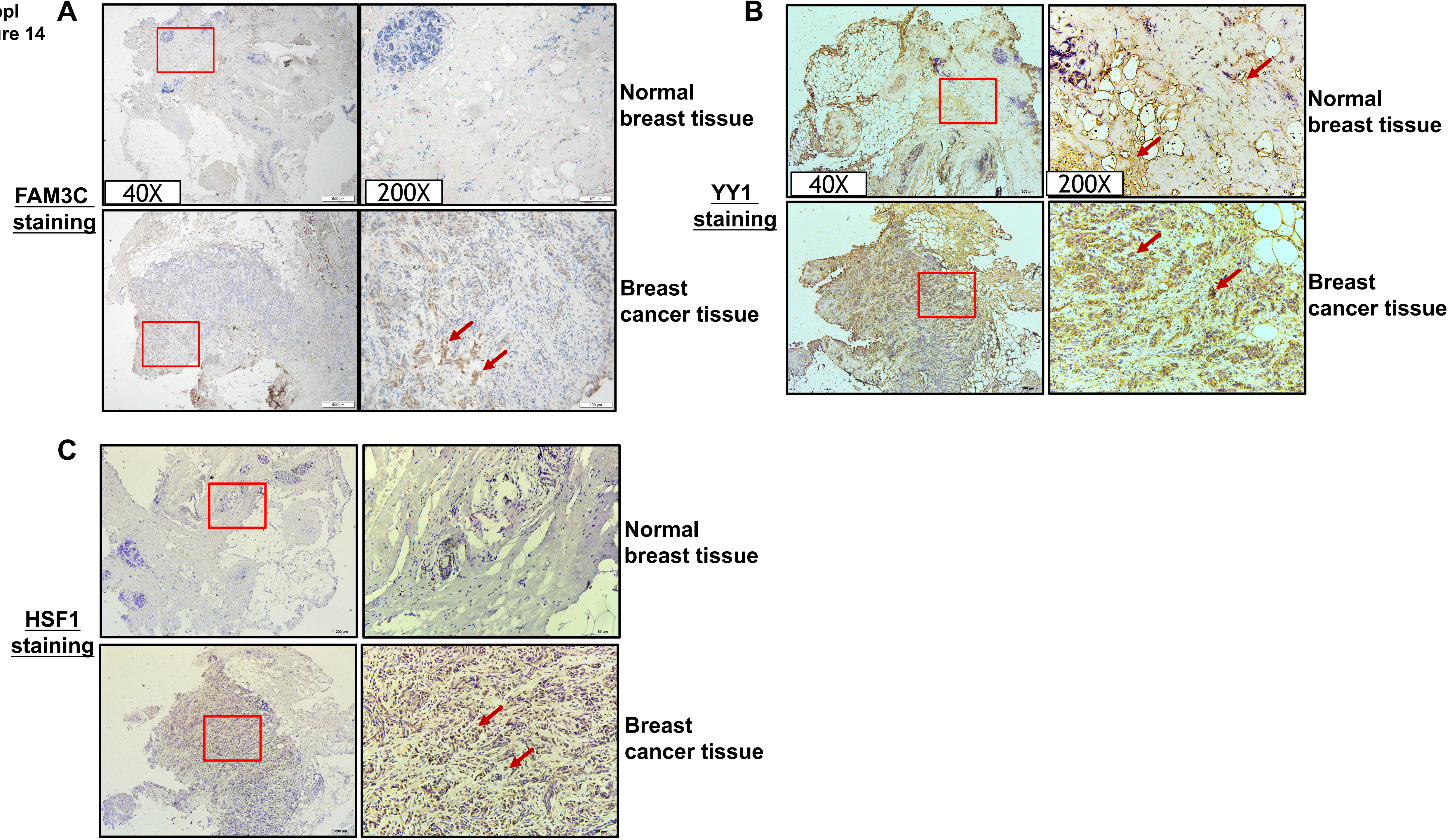

Supplement: Supplementary file 1 [file JCMM-23-3464-s001.pdf]
