# Supplementary material for: FAM3C‐YY1 axis is essential for TGFβ‐promoted proliferation and migration of human breast cancer MDA‐MB‐231 cells via the activation of HSF1
Source: J Cell Mol Med. 2019 Mar 19;23(5):3464–75. doi: 10.1111/jcmm.14243 (PMC6484506; doi:10.1111/jcmm.14243)
Supplement: Supplementary file 2 [file JCMM-23-3464-s002.doc]

**Supplemental table 1. siRNA sequence against human FAM3C and YY1 mRNAs**

| Dulex Name | SenseSeq | AntiSeq |
| --- | --- | --- |
| FAM3C-1(H) | GGAUUAUCCCUCAACUAUATT | UUCAUUAAUAUGCUCCGGCTT |
| FAM3C-2(H) | GUUGGAAGCUAUUCGGAUATT | UAUCCGAAUAGCUUCCAACTT |
| FAM3C-3(H) | AGCGCACUUUCACUCUUAATT | UUAAGAGUGAAAGUGCGCUTT |
| FAM3C-4(H) | CGAUGAUGGAGCAACCAAATT | UUUGGUUGCUCCAUCAUCGTT |
| YY1-1(H) | CAUGGAUCGUGGUCAGCAUTT | AUGCUGACCACGAUCCAUGTT |
| YY1-2(H) | AGAUCUGCCUCGAGGACAATT | UUGUCCUCGAGGCAGAUCUTT |
| YY1-3(H) | GGCUGUAUCCCGCGGAGAATT | UUCUCCGCGGGAUACAGCCTT |
| YY1-4(H) | UGGUGUUCGUGGCAUCCUATT | UAGGAUGCCACGAACACCATT |

H:Human

**Supplemental table 2**. List of oligonucleotide primer pairs used in real time RT-PCR analysis.

| Name | Pubmed accession number | Forward | Reverse |
| --- | --- | --- | --- |
| FAM3C(H) | NM_014888.2 | 5'-GGAGATGTGGCACCATTTATTG -3' | 5'-GAGTTTGGTTGCTCCATCATC -3' |
| HSF1(H) | NM_005526.3 | 5'-ATAGCCCCAGTAGGACAAACG -3' | 5'- TGTGAAGCCCCAACCAAAC -3' |
| Cyclin D1(H) | NM_053056.2 | 5’- AGCTGTGCATCTACACCGAC -3’ | 5’-GAAATCGTGCGGGGTCATTG -3’ |
| YY1(H) | NM_003403.4 | 5’-CACACAGAGGGAAGACCAGGC -3’ | 5’-AAACCAAATAACCAACGACCA-3’ |
| GAPDH(H) | NM_001256799.2 | 5’- AATGGGCAGCCGTTAGGAAA -3’ | 5’- GCGCCCAATACGACCAAATC -3’ |

H: Human

**Supplemental figure legends**

**Figure S1. TGFβ upregulated FAM3C and HSF1 expression in MDA-MB-231 cells**. A) TGFβ increased cell viability. Cells were treated with different concentrations of TGFβ for 24 hours. B) TGFβ increased FAM3C and HSF1 mRNA levels. Cells were treated with 2ng/μl TGFβ for 24 hours. C-D) TGFβ increased FAM3C and HSF1 protein levels. Cells were treated with 2ng/μl TGFβ for 24 hours. Representative gel images were shown in panel C, and quantitative data shown in panel D. N=4-6, *P<0.05 versus control cells.

**Figure S2. FAM3C overexpression promoted migration of MDA-MB-231 cells.** A) FAM3C overexpression promoted migration of breast cancer cells as evaluated by determining the moving distance. Representative images shown in upper panel, and quantitative data shown in lower panel. B) FAM3C overexpression promoted migration of breast cancer cells as evaluated by wound area calculation. Representative images shown in upper panel, and quantitative data shown in lower panel. N=4, *P<0.05 versus Ad-GFP-treated cells.

**Figure S3. FAM3C silencing inhibited TGFβ-promoted migration of MDA-MB-231 cells.** FAM3C silencing inhibited TGFβ-induced migration of breast cancer cells as evaluated by moving area calculation. Representative images shown in upper panel, and quantitative data shown in lower panel. N=4, *P<0.05 versus scramble group of cells, #P<0.05 versus scramble+TGFβ group of cells.

**Figure S4. HSF1 inhibition repressed TGFβ-promoted migration of MDA-MB-231 cells.** HSF1 inhibition blocked TGFβ-induced migration of breast cancer cells as evaluated by wound area calculation. Representative images shown in upper panel, and quantitative data shown in lower panel. N=3, *P<0.05 versus control cells, #P<0.05 versus TGFβ group of cells.

**Figure S5. Plasmid HSF1 overexpression activated Akt and promoted proliferation of MDA-MB-231 cells.** A) HSF1 plasmid transfection on the protein levels of HSF1 and pAkt. Representative gel images shown in upper panel, and quantitative data shown in lower panel. B) Representative images of cell density after HSF1 overexpression. C) Cell number counting assays after HSF1 overexpression. D) Cell viability assays after HSF1 overexpression. KRI, HSF1 plasmid-transfected cells treated with HSF1 inhibitor KRIBB11. N=3-5, *P<0.05 versus pGFP-treated cells, #P<0.05 versus pHSF1-trreated cells.

**Figure S6. Prediction of potential transcriptor binding sites in the promoter of human HSF1 gene**. The binding sites in human HSF1 gene promoter were analyzed using UCSC Genome Browser and TRANSFAC® 7.0 Public in the following website: http://www.gene-regulation.com/pub/databases.html. Some certain potential sites with the highest prediction scores were listed. 6 potential binding sites highly specific for transcription factor YY1 were predicted to be existed in the promoter region of human HSF1 gene.

**Figure S7. Inhibition of HSF1 repressed YY1-induced migration of MDA-MB-231 cells.** Inhibition of HSF1 repressed YY1-induced migration of cells was evaluated by wound are calculation. Representative images shown in left panel, and quantitative data shown in right panel. N=3, *P<0.05 versus pGFP group of cells, #P<0.05 versus pYY1-treated cells.

**Figure S8. Silencing of YY1 repressed TGFβ-induced HSF1 upregulation and proliferation of MDA-MB-231 cells.** A) Silencing of YY1 repressed TGFβ-induced HSF1 upregulation and Akt activation. Representative gel images shown in upper panel, and quantitative data shown in lower panel. B) Silencing of YY1 on TGFβ-induced change in HSF1 and Cyclin D1 mRNA levels. C) Representative images of cell density after YY1 inhibition. D) Cell number counting assays after YY1 inhibition. E) Cell viability assays after YY1 inhibition. N=3-5, *P<0.05 versus scramble group of cells, #P<0.05 versus scramble+TGFβ group of cells.

**Figure S9. YY1 silencing inhibited TGFβ-promoted migration of MDA-MB-231 cells.** YY1 silencing inhibited TGFβ-induced migration of breast cancer cells as evaluated by wound area calculation. Representative images shown in upper panel, and quantitative data shown in lower panel. N=4, *P<0.05 versus scramble group of cells, or between two indicated two groups of cells.

**Figure S10. TGFβ and FAM3C activated YY1-HSF1-Akt signaling axis in BT-549 cells**. A-B) TGFβ treatment increased the mRNA (A) and protein (B) levels of FAM3C, YY1 and HSF1 in BT-549 cells. Cells were treated with 2ng/μl of TGFβ for 24 hours. C-D) FAM3C overexpression upregulated the mRNA (C) and protein (D) levels of YY1 and HSF1 in BT-549 cells. Cells were infected with Ad-GFP or Ad-FAM3C for 24 hours. In panels B and D, representative gel images were shown in upper panel, and quantitative data shown in lower panel. N=5, *P<0.05 versus corresponding control cells.

**Figure 11. Inhibition of HSF1 repressed FAM3C-induced proliferation of BT-549 cells.** HSF1 inhibition blocked FAM3C-induced proliferation of BT-549 cells. Representative cell images were shown in left panel, and cell counting number data shown in right panel D. N=5, *P<0.05 between two indicated groups of cells.

**Figure 12. Inhibition of HSF1 inhibited TGFβ-induced proliferation of BT-549 cells.** HSF1 inhibition repressed TGFβ-promoted proliferation of BT-549 cells. Representative cell images were shown in left panel, and cell counting number data shown in right panel. N=5, *P<0.05 between two indicated groups of cells.

**Figure S13. Inhibition of HSF1 blocked FAM3C- and TGFβ-induced migration of BT-549 cells.** A) HSF1 inhibition blocked FAM3C-induced migration of BT-549 cells. B) HSF1 inhibition blocked TGFβ-promoted migration of BT-549 cells. Representative images were shown in left panels and quantitative data shown in right panels. N=4, *P<0.05 versus control cells, #P<0.05 versus TGFβ- or FAM3C-treated cells.

**Figure S14. FAM3C-YY1-HSF1 axis was activated in human breast cancer tissue.** A-C) Immunohistochemical staining revealed that FAM3C (A), YY1 (B) and HSF1 (C) protein levels were increased in breast cancer tissues when compared normal breast tissues. Additional slides from another breast cancer tissue and its corresponding adjacent normal tissue were stained with FAM3C, YY1 and HSF1 antibodies, respectively. Positive cells were stained in brown, and representative positive cells were indicated by arrows in the images. The amplification power had been marked in the images.
